# Lkb1 controls brown adipose tissue growth and thermogenesis by regulating the intracellular localization of CRTC3

**DOI:** 10.1038/ncomms12205

**Published:** 2016-07-27

**Authors:** Tizhong Shan, Yan Xiong, Pengpeng Zhang, Zhiguo Li, Qingyang Jiang, Pengpeng Bi, Feng Yue, Gongshe Yang, Yizhen Wang, Xiaoqi Liu, Shihuan Kuang

**Affiliations:** 1 Department of Animal Sciences, Purdue University, West Lafayette, Indiana 47907, USA; 2 College of Animal Sciences, Zhejiang University, Hangzhou, Zhejiang 310058, China; 3 Laboratory of Animal Fat Deposition and Muscle Development, College of Animal Science and Technology, Northwest A&F University, Yangling 712100, China; 4 Department of Biochemistry, Purdue University, West Lafayette, Indiana 47907, USA; 5 Purdue University Center for Cancer Research, West Lafayette, Indiana 47907, USA

## Abstract

Brown adipose tissue (BAT) dissipates energy through Ucp1-mediated uncoupled respiration and its activation may represent a therapeutic strategy to combat obesity. Here we show that Lkb1 controls BAT expansion and UCP1 expression in mice. We generate adipocyte-specific Lkb1 knockout mice and show that, compared with wild-type littermates, these mice exhibit elevated UCP1 expression in BAT and subcutaneous white adipose tissue, have increased BAT mass and higher energy expenditure. Consequently, KO mice have improved glucose tolerance and insulin sensitivity, and are more resistant to high-fat diet (HFD)-induced obesity. Deletion of Lkb1 results in a cytoplasm to nuclear translocation of CRTC3 in brown adipocytes, where it recruits C/EBPβ to enhance Ucp1 transcription. In parallel, the absence of Lkb1 also suppresses AMPK activity, leading to activation of the mTOR signalling pathway and subsequent BAT expansion. These data suggest that inhibition of Lkb1 or its downstream signalling in adipocytes could be a novel strategy to increase energy expenditure in the context of obesity, diabetes and other metabolic diseases.

## Abstract (web summary)

The kinase Lkb1 is expressed in various metabolic tissues and is known to regulate cellular and systemic energy homeostasis. Here, the authors delete Lkb1 specifically in mature adipocytes of mice to show that Lkb1 regulates brown adipose tissue expansion and expression of UCP1.

## Graphical abstract

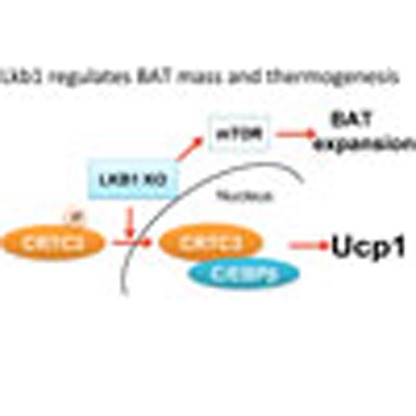

The global epidemic of obesity is associated with high risks of metabolic diseases including type 2 diabetes, insulin resistance, heart disease, stroke, hypertension and cancer^1^. When energy intake consistently exceeds total energy expenditure, the excessive energy is stored in white adipocytes, whose expansion results in obesity. Adipose tissues also play an important role in energy expenditure^2^. Brown adipose tissue (BAT) is specialized in breaking down lipids to generate heat to defend against hypothermia. This thermogenic property of BAT is mediated by Ucp1, a mitochondria protein that uncouples electron transport from ATP production, leading to generation of heat^2^,^3^,^4^. Recently, a population of Ucp1-expressing thermogenic cells was also identified in WAT. These so-called beige or brite (brown in white) adipocytes can be induced by sympathetic nerve innervation, cold exposure, chemical (or hormonal) stimulations and alterations in gene expression^1^,^5^,^6^,^7^,^8^,^9^,^10^,^11^,^12^,^13^,^14^,^15^,^16^. Beige adipocytes are similar to classical brown adipocytes in that they can burn lipids to produce heat, but can be distinguished from classical brown adipocytes by the expression of specific cell surface markers (Tmem26 and CD137)^2^.

The thermogenic activity of brown and beige adipocytes has been reported to increase energy expenditure and thus counteracts obesity^5^,^17^,^18^,^19^. In contrast, transgenic mice that are genetically ablated of BAT are prone to the development of obesity^20^. Notably, beige and brown adipocytes have recently been detected in adult humans^21^,^22^,^23^,^24^,^25^,^26^,^27^. The prevalence of beige or brown adipocytes in adult human is inversely correlated with body mass index, adiposity and fasting plasma glucose level^24^, indicating these UCP1-expressing adipocytes play an important role in regulating metabolism. Moreover, activation of BAT/beige adipocytes thermogenesis by cold exposure, or by β3-adrenergic receptor agonist activation, has been linked to increased energy expenditure, reduced adiposity and lower plasma lipids^21^,^25^,^28^. Therefore, understanding the molecular regulation of brown and beige adipocyte activity and biogenesis may lead to novel strategies to control energy homoeostasis.

The serine/threonine kinase 11 (Stk11), commonly known as liver kinase b1 (Lkb1), is initially identified as a tumour suppressor mutated in Peutz–Jeghers syndrome^29^,^30^. Lkb1 plays important roles in various biological processes including cellular energy metabolism^31^, cell polarity^32^ and cancer initiation and progression^33^. Tissue-specific deletions of *Lkb1* in the liver^34^, pancreas^32^,^35^, heart^36^ and skeletal muscle^37^,^38^,^39^,^40^ have revealed that Lkb1 functions to control glucose homoeostasis and energy metabolism in various tissues. Recently, Zhang *et al.*^41^ generated *Fabp4-Cre* mediated Lkb1 knockout (KO) mouse model. Interestingly, *Fabp4-Cre* failed to induce Lkb1 deletion in BAT in that model^41^, thus the role of Lkb1 in BAT has yet to be determined. In this study, we used a mature adipocyte-restricted *adiponectin-Cre* (abbreviated as *Adipoq-Cre*) mouse model^42^,^43^ to specifically delete Lkb1 in WAT and BAT. We found that *Adipoq-Cre* induces efficient deletion of Lkb1 in various adipose tissues including BAT, and results in robust metabolic phenotypes. We further elucidated the molecular mechanisms underlying the role of Lkb1 in BAT. Our results demonstrate that Lkb1 is a critical regulator of BAT growth and function, and suggest that Lkb1 signalling may be therapeutically targeted to counteract obesity, diabetes and other metabolic diseases.

## Results

### Adipocyte-specific deletion of Lkb1 expands BAT

**Figure 1:**
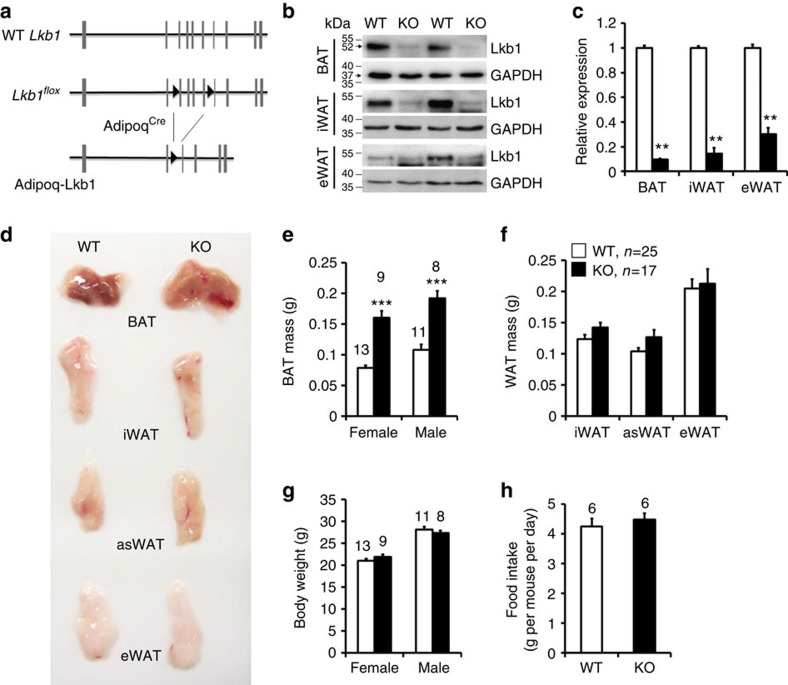
*Adipoq-Cre*-mediated deletion of Lkb1 increases the mass of BAT but not WAT. (**a**) Targeting strategy for adipocyte-specific deletion of *Lkb1*. Vertical lines represent exons and triangles represent LoxP. (**b**,**c**) Efficient reduction of Lkb1 protein (**b**) and mRNA (**c**, *n*=4) levels in BAT and WAT depots of the *Adipoq-Lkb1* KO mice. (**d**) Representative images of BAT and WAT depots showing specific enlargement of Lkb1 KO BAT. (**e**–**h**) Lkb1 deletion increases the weights of BAT (**e**) without affecting WAT (**f**, WT *n*=25, KO *n*=17) and overall body weight (**g**), or food intake (**h**). Numbers above the bars are animals (2–3 months old) analysed. Error bars, s.e.m., **P<0.01, ***P<0.001, two-tailed Student's *t*-test.

To directly investigate the role of Lkb1 in adipose tissues, we used the Cre-loxP recombination system involving *Adipoq-Cre* and *Lkb1*^*flox/flox*^ mice (Fig. 1a). Previous studies verified the adipocyte-specific expression pattern of *Adipoq-Cre*^42^,^43^. Thus, in the *Adipoq-Cre*/ *Lkb1*^*flox/flox*^ mice (abbreviated as *Adipoq-Lkb1*) all adipocytes should be deleted of exons 3–6 of the *Lkb1* gene, leading to loss of the kinase domain and premature translational termination of the Lkb1 protein (Fig. 1a). Western blot and quantitative PCR analysis confirmed the efficient and specific deletion of Lkb1 in BAT and WAT (Fig. 1b,c), but not in non-adipose tissues including spleen, lung, kidney, liver, heart and muscle (Supplementary Fig. 1a).

The *Adipoq-Lkb1* mice were born at expected Mendelian ratio, and morphologically indistinguishable from their wild-type (WT) littermates. Notably, the BAT size of the female and male *Adipoq-Lkb1* mice was substantially enlarged: equivalent to 204 and 177% of the BAT weight in sex-matched WT mice, respectively (Fig. 1d,e). The size and weight of various WAT depots, however, were identical in the WT and *Adipoq-Lkb1* mice (Fig. 1d,f). In addition, the weights of other non-adipose tissues were not affected by *Adipoq-Lkb1* deletion (Supplementary Fig. 1b,c). When fed on regular chow diet, the *Adipoq-Lkb1* mice had similar body weights, growth curves and energy intakes as the WT littermates (Fig. 1g,h). Taken together, deletion of Lkb1 in adipocytes caused a specific expansion of BAT but not WAT.

**Figure 2:**
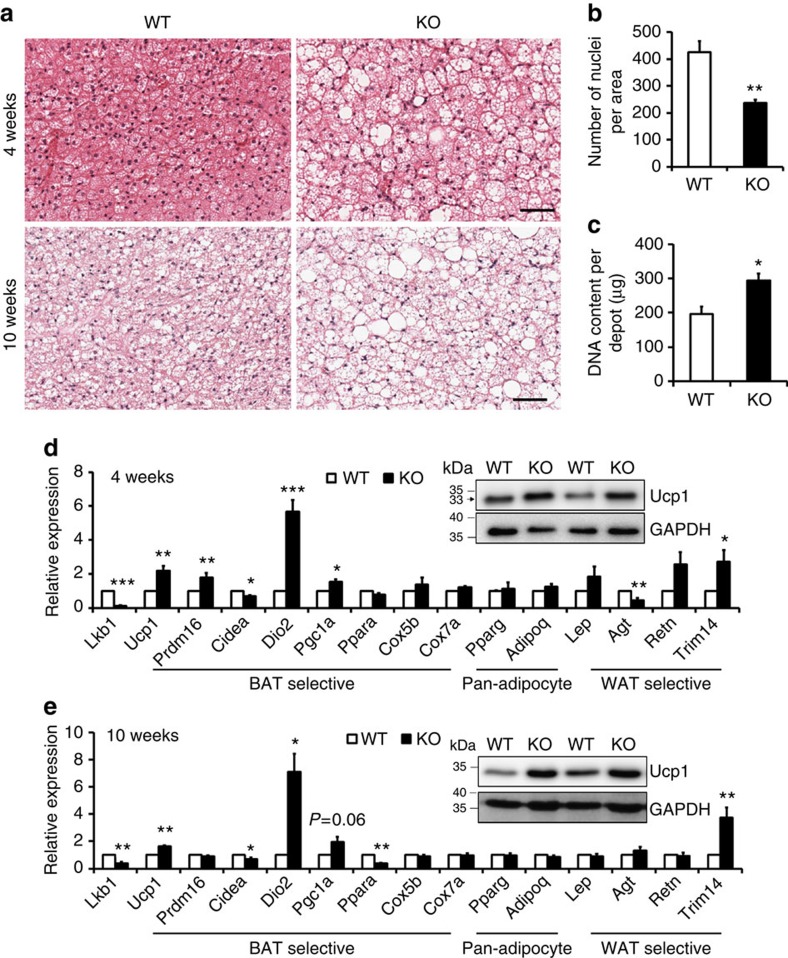
Lkb1 deficiency leads to BAT hypertrophy and hyperplasia and increases the expression of BAT signature genes. (**a**) H&E staining of BAT sections from WT (*Lkb1*^*flox/flox*^) and KO (*Adipoq-Lkb1*) mice at 4 and 10 weeks old. Scale bars, 100 μm. (**b**) Number of nuclei per BAT image in WT (*n*=9) and KO (*n*=11) mice, from each mouse two random images were counted. (**c**) Genomic DNA content per BAT depot of WT (*n*=3) and KO (*n*=4) mice at 10 weeks old. (**d**,**e**) mRNA of pan-adipocyte, BAT- and WAT-selective genes in BAT from WT and KO mice at 4 weeks old (**d**, *n*=7) and 10 weeks old (**e**, *n*=7). Insets are western blots showing Ucp1 protein levels in BAT. Error bars represent s.e.m. *P<0.05, **P<0.01, ***P<0.001, two-tailed Student's *t*-test.

We next examined whether the increased BAT mass in *Adipoq-Lkb1* mice is due to hypertrophy (increase in adipocyte size) or hyperplasia (increase in adipocyte number). Hematoxylin-eosin (H&E) staining revealed an obvious increase in adipocyte size in *Adipoq-Lkb1* BAT compared with WT BAT, accompanied by increased abundance of lipid droplets and infiltration of oligolocular and unilocular adipocytes (Fig. 2a). Nuclei densities (nuclei number per unit area) were also significantly lower in the *Adipoq-Lkb1* mice compared with the WT mice (Fig. 2a,b), confirming larger adipocyte size in the KO mice. In addition, genomic DNA content per BAT depot was higher in the *Adipoq-Lkb1* mice compared with the WT mice (Fig. 2c), suggesting that the *Adipoq-Lkb1* BAT contains more cells per depot than the WT BAT depots. To investigate whether increased cell proliferation contributes to the hyperplasia, we used Ki67 staining to mark proliferating cells. At P2 stage when BAT undergoes fast growth, the *Adipoq-Lkb1* BAT contains a higher percentage of Ki67^+^ cells than WT BAT (Supplementary Fig. 2a). This observation suggests that Lkb1 deletion in post-mitotic adipocytes may exert paracrine or contact-dependent effects on progenitor cell proliferation. These data indicated that expansion of BAT mass in the *Adipoq-Lkb1* mice is a consequence of combined hypertrophy and hyperplasia of adipocytes.

### Lkb1 KO upregulates BAT-specific genes

We further analysed the expression of a panel of adipogenic genes. Despite the apparent infiltration of unilocular white adipocyte-like cells, the *Adipoq-Lkb1* BAT expressed higher levels of Ucp1 than the WT BAT (Fig. 2d,e). Consistently, the mRNA levels of BAT-selective *Prdm16*, *Dio2* and *Pgc1a* genes were significantly higher in the *Adipoq-Lkb1* BAT than the WT BAT (Fig. 2d,e). The mRNA levels of the pan-adipocyte genes *Pparg* and *Adipoq* were indistinguishable between the two genotypes (Fig. 2d,e). Consistent with the observed infiltration of unilocular adipocytes, the expression of WAT-selective *Trim14* gene was also higher in the *Adipoq-Lkb1* than WT BAT, but the expression of *Agt* and *Retn*, two other WAT-selective genes, was not affected (Fig. 2d,e). These data indicate that the expansion of BAT in *Adipoq-Lkb1* mice is associated with higher expression levels of BAT-selective genes.

To investigate whether deletion of Lkb1 affects brown adipocyte differentiation in culture, we isolated SVF cells from BAT depots of WT and *Adipoq-Lkb1* mice. Lkb1 deletion marginally promoted brown adipocytes differentiation and triglyceride (TG) accumulation (Supplementary Fig. 3a,b). Consistent with the *in vivo* data, Lkb1 deficiency markedly increased the expression of *Ucp1* and *Pgc1a* in cultured brown adipocytes (Supplementary Fig. 3c,d). By contrast, overexpression of Lkb1 suppressed brown adipocyte differentiation and TG accumulation (Supplementary Fig. 4a–c), accompanied by apparent inhibition of *Prdm16, Pgc1a, Ucp1, Pparg, C/ebpa* and *Fabp4* expression (Supplementary Fig. 4d). Taken together, these results indicate that deletion of Lkb1 promotes brown adipocyte proliferation, differentiation and gene expression *in vivo* and *in vitro*.

### Deletion of Lkb1 induces browning of subcutaneous WAT

We next investigated how deletion of Lkb1 affects WAT depots. Although the appearance and mass of inguinal WAT (iWAT), anterior subcutaneous WAT (asWAT) and epididymal WAT (eWAT) were indistinguishable between the *Adipoq-Lkb1* and WT mice (Fig. 1), H&E staining revealed that the *Adipoq-Lkb1* iWAT contains numerous small multilocular cells resembling beige adipocytes (Supplementary Fig. 5a). However, the genomic DNA contents per iWAT depot were identical in WT and *Adipoq-Lkb1* mice (Supplementary Fig. 5b). These results suggest that *Lkb1* deletion does not alter the overall number and size of white adipocytes.

Consistent with the appearance of multilocular cells in iWAT, the beige/brown adipocyte-specific Ucp1 protein was expressed at much higher levels in the subcutaneous WAT depots of *Adipoq-Lkb1* than those of WT mice (Supplementary Fig. 5c,d). Furthermore, compared with the WT counterparts, *Adipoq-Lkb1* iWAT and asWAT expressed higher mRNA levels of BAT signature markers *Ucp1* and *Pgc1a*, but not WAT markers *Adipoq* and *Lep* (Supplementary Fig. 5c,d). Interestingly, deletion of Lkb1 did not affect the expression of Ucp1 in eWAT (Supplementary Fig. 5e). These data suggest that lack of Lkb1 robustly induces browning of subcutaneous WAT but not visceral WAT.

Cold stress stimulates beige adipocytes formation and browning of WAT^16^,^25^. The rectal temperatures of the *Adipoq-Lkb1* mice were higher than those of WT mice after cold exposure for 3 h, even though they had identical rectal temperatures at the room temperature (Supplementary Fig. 5f). In addition, cold exposure elicited a more profound induction of Ucp1 expression in iWAT of *Adipoq-Lkb1* mice compared with the WT mice (Supplementary Fig. 5g). The mRNA levels of *Ucp1* and *Pgc1a* were eight- and six-fold higher, respectively, in the iWAT of *Adipoq-Lkb1* mice compared with WT mice after cold exposure (Supplementary Fig. 5h). These results indicate that *Adipoq-Lkb1* mice are more adaptive to cold-induced browning.

We further analysed SVF cells from iWAT of WT and *Adipoq-Lkb1* mice. Deletion of Lkb1 promoted adipogenic differentiation and TG accumulation, as revealed by Oil red O staining (Supplementary Fig. 6a,b). Notably, the protein level of Ucp1 was significantly higher in Lkb1 KO than WT adipocytes (Supplementary Fig. 6c). The mRNA levels of the browning markers *Ucp1*, *Cidea*, *Pgc1a* and *Ppara*, were increased by 62-, 10-, 4- and 4-fold, respectively, in the Lkb1 KO adipocytes compared with the WT cells (Supplementary Fig. 6d). Conversely, overexpression of Lkb1 inhibited differentiation of WAT SVF cells and TG accumulation (Supplementary Fig. 7a–c), accompanied by apparent inhibition of *Ucp1, Pparg, C/ebpa* and *Fabp4* expression, but not *Prdm16* and *Pgc1a* expression (Supplementary Fig. 7d). These results indicate that the deletion of Lkb1 promotes browning of WAT in a cell autonomous manner.

### Improved glucose metabolism in *Adipoq-Lkb1* mice

**Figure 3:**
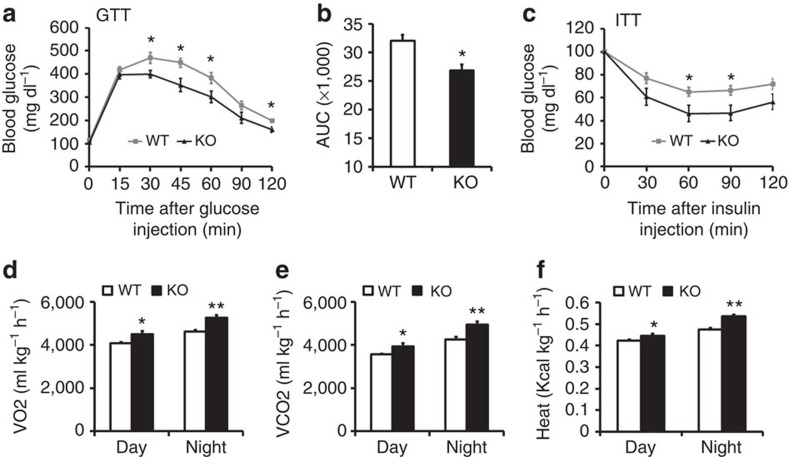
Improved glucose tolerance and insulin sensitivity and higher metabolic rate in *Adipoq-Lkb1* KO mice. (**a**) Blood glucose concentrations during glucose tolerance tests (GTT) performed on 6-week-old WT and KO male mice (*n*=7). (**b**) Area under curve (AUC) calculated based on data in **a**. (**c**) Blood glucose concentrations during insulin tolerance tests (ITT) performed on 10-week-old WT and KO male mice (*n*=8). (**d**–**e**) Average day and night O2 consumption (VO2, **d**), CO2 production (VCO2, **e**) and heat production (**f**). *n*=4. Error bars represent s.e.m. *P<0.05, **P<0.01, two-tailed Student's *t*-test.

As brown and beige adipocytes improve glucose metabolism and insulin sensitivity^19^,^44^, we next examined whether expansion of BAT and browning of WAT in the *Adipoq-Lkb1* mice elicits beneficial metabolic effects. We conducted glucose tolerance tests (GTT) and insulin tolerance tests (ITT). Compared with the WT littermates, *Adipoq-Lkb1* mice had lower blood glucose levels after intraperitoneal (i.p.) injection of glucose (Fig. 3a). The area under curve of the Lkb1 KO mice was significantly smaller than those of WT mice (Fig. 3b). We also observed faster insulin-stimulated glucose clearance in *Adipoq-Lkb1* mice compared with the WT mice (Fig. 3c). In addition, the *Adipoq-Lkb1* mice had higher rates of oxygen consumption and carbon dioxide production, and expended more energy compared with the WT mice (Fig. 3d–f; Supplementary Fig. 8a–c). Taken together, these results suggest that adipocyte-specific deletion of Lkb1 improves systemic insulin sensitivity, glucose tolerance and energy metabolism. However, we can't exclude the possibility that alterations in physical activity may have also partially contributed to the higher energy expenditure, despite similar motor behaviours being observed (Supplementary Movie 1).

### *Adipoq-Lkb1* mice are resistant to HFD-induced obesity

**Figure 4:**
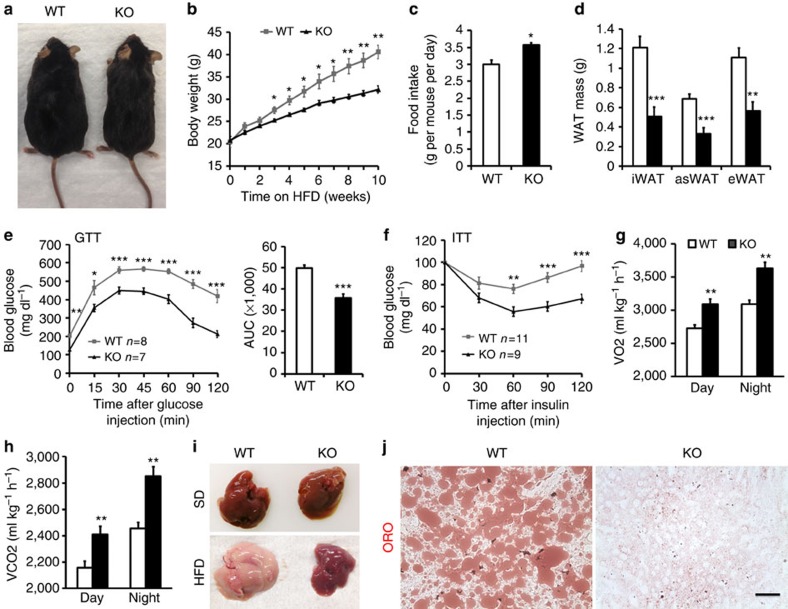
Adipocyte-specific deletion of Lkb1 protects mice against high-fat diet (HFD)-induced obesity. (**a**) Representatives of WT (*Lkb1*^*flox/flox*^) and KO (*Adipoq-Lkb1*) mice fed with HFD for 12 weeks. (**b**,**c**) Growth curve (**b**) and feed intake (**c**) of WT (*n*=6) and KO (*n*=7) mice during HFD feeding. (**d**) WAT mass of WT (*n*=6) and KO (*n*=7) mice after 12 weeks on HFD. (**e**,**f**) GTT (**e**) and ITT (**f**) curves of WT and KO mice after 10 weeks on HFD. (**g**,**h**) Average day and night O2 consumption (VO2, **g**) and CO2 production (VCO2, **h**) of WT (*n*=9) and KO (*n*=8) mice after 10-week HFD. (**i**) Representative images of whole livers from WT and KO mice fed with a standard diet (s.d.) or HFD for 12 weeks. (**j**) Liver sections stained with Oil Red O after 12 weeks on HFD. Scale bars, 100 μm. Error bars represent s.e.m. *P<0.05, **P<0.01, ***P<0.001, two-tailed Student's *t*-test.

To examine the long-term effect of Lkb1 deletion on energy metabolism, we fed the mutant mice and WT littermates with high-fat diet (HFD). The *Adipoq-Lkb1* mice appeared much leaner than the WT littermates after HFD feeding (Fig. 4a). The body weight of *Adipoq-Lkb1* mice, which was similar to WT mice before HFD feeding, was consistently lighter than the WT mice after 3 weeks on HFD (Fig. 4b), even though larger amounts of food were consumed (Fig. 4c). After fed with HFD for 12 weeks, the average body weight of WT was 44.8±1.8 g, while that of the *Adipoq-Lkb1* mice was only 33.7±1.0 g (Supplementary Fig. 9a). The body weight difference was mainly due to a reduction of adipose and liver mass in the *Adipoq-Lkb1* mice (Fig. 4d; Supplementary Fig. 9b,c). Compared with WT mice, the *Adipoq-Lkb1* mice retained better glucose tolerance and higher insulin sensitivity after HFD feeding (Fig. 4e,f). The fasting glucose levels of the *Adipoq-Lkb1* mice were also lower than that of WT mice (Supplementary Fig. 9d–h). In addition, the *Adipoq-Lkb1* mice retained higher respiration rate and energy expenditure after HFD treatment (Fig. 4g,h). Furthermore, the lipid content was much lower in liver of *Adipoq-Lkb1* mice than in WT mice, especially after HFD feeding (Fig. 4i,j; Supplementary Fig. 9i). These results together suggest that BAT expansion and the browning of WAT in *Adipoq-Lkb1* mice increases energy expenditure and protects mice from HFD-induced obesity and fatty liver.

To examine whether the systemic metabolic changes were due to enhance energy expenditure of adipocytes, we analysed oxygen consumption rate (OCR) of adipocytes differentiated from *Adipoq-Lkb1* and WT SVF cells. The basal respiration, uncoupled respiration after Oligomycin inhibition of ATP synthesis and maximal respiration after stimulation with carbonyl cyanide 4-(trifluoromethoxy)phenylhydrazone (FCCP) were all significantly higher in *Adipoq-Lkb1* brown adipocytes than in WT adipocytes (Supplementary Fig. 10a,b). Likewise, *Adipoq-Lkb1* iWAT adipocytes exhibited higher basal and uncoupled OCR (Supplementary Fig. 10c,d).

To investigate whether the improved energy expenditure of *Adipoq-Lkb1* mice requires Ucp1 function, we analysed mice fed on a HFD and housed at 30 °C (thermoneutrality) to block Ucp1 function. Interestingly, the body weights of Lkb1 KO mice were similar to the WT mice until 7 weeks after HFD feeding (Supplementary Fig. 11a,b). In addition, WT and *Adipoq-Lkb1* mice had similar glucose tolerance and insulin sensitivity (Supplementary Fig. 11c,d). Furthermore, thermoneutrality treatment abolished the beneficial metabolic phenotypes of *Adipoq-Lkb1* mice observed at room temperature (Supplementary Fig. 11e–g). However, there is a non-significant trend that the *Adipoq-Lkb1* mice still maintain a lower body weight, better glucose tolerance and insulin sensitivity (Supplementary Fig. 11b–d). Given this observation, we conclude that the higher energy expenditure in the *Lkb1 KO* mice is predominantly, but not exclusively, Ucp1 dependent. Other factors such as skin, fur and tail that influencing cold perception may also play a role^45^, even though we did not detect any morphological abnormality in skin, fur and tail in the Lkb1 KO mice.

### Lkb1 KO induces BAT expansion through AMPK-mTOR pathway

To investigate how Lkb1 deficiency leads to the expansion of BAT, we examined the canonical Lkb1-AMPK-mTOR pathway involved in controlling cell growth. Deletion Lkb1 inhibited the kinase activity of AMPK in BAT (Supplementary Fig. 12a) without altering the activity of SIK (Supplementary Fig. 12a), another Lkb1 substrate that has been shown to regulate adipogenesis and insulin signalling^46^,^47^. In addition, Lkb1 deletion reduced the levels of phosphorylated AMPK (pAMPK, T172) and increased phosphorylated S6 (pS6, S240/244) in BAT (Supplementary Fig. 12b), suggesting activation of the mTOR pathway.

As mTOR activation promotes adipogenesis and inactivation reduces fat mass^48^,^49^, we further examined whether Lkb1 regulate BAT size through the mTOR pathway. To test this, we established the *Adipoq-Cre/ Lkb1*^*flox/flox*^*/ mTOR*^*flox/flox*^ mice (adipocyte-specific *Lkb1* and *mTOR* double KO mice, abbreviated as DKO). The body weights of WT, *Adipoq-Lkb1* and DKO were similar (Supplementary Fig. 12c) but the BAT expansion phenotype in *Adipoq-Lkb1* is rescued by DKO (Supplementary Fig. 12d–f). Furthermore, DKO rescued or reversed the expression pattern of adipogenic and BAT-selective genes in *Adipoq-Lkb1* BAT (Supplementary Fig. 12g). These results suggest that deletion of Lkb1 promotes BAT expansion through dampening AMPK activity and activating the mTOR pathway.

### Lkb1 deletion induces nuclear translocation of CRTC3

**Figure 5:**
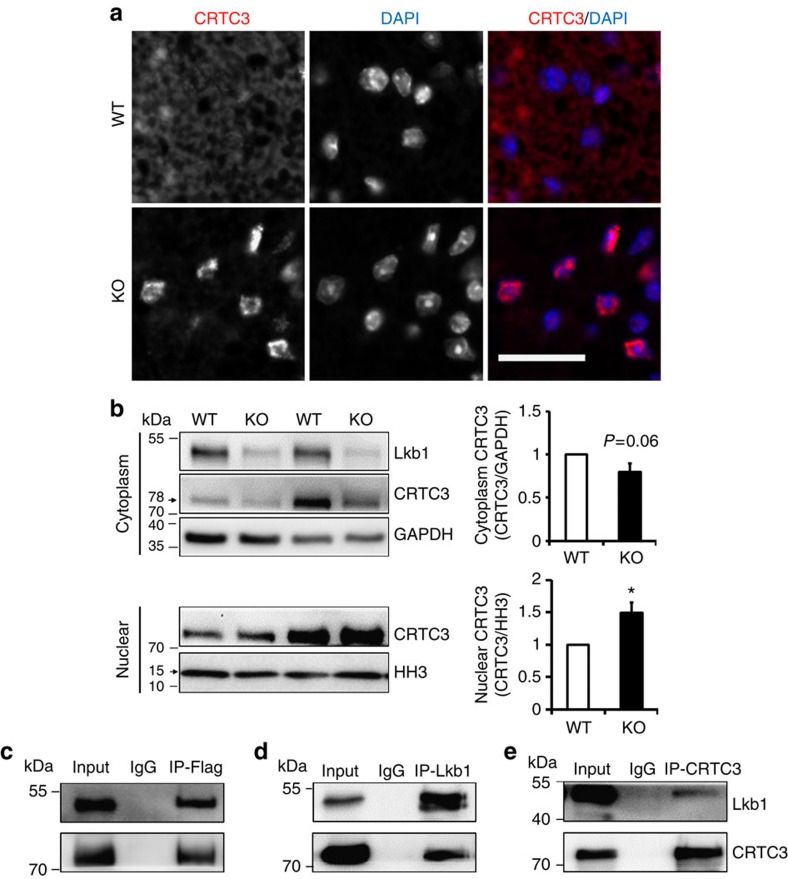
Lkb1 interacts with CRTC3 to affect its nuclear translocation. (**a**) Localization of CRTC3 to the nucleus (labelled by DAPI) in BAT sections of Lkb1 KO (*Adipoq-Lkb1*) but not WT (*Lkb1*^*flox/flox*^) mice. Scale bars, 25 μm. (**b**) CRTC3 protein abundance in the cytoplasmic (*n*=4) and nuclear fractions (*n*=6) of BAT from WT and KO mice. Error bars represent s.e.m. *P<0.05, two-tailed Student's *t*-test. (**c**–**e**) Lkb1 interacts with CRTC3. 293T cells were transfected with pCDNA-Flag-CRTC3 and the lysates were immunoprecipitated (IP) with Flag (**c**), Lkb1 (**d**) and CRTC3 (**e**) antibodies and blotted with Lkb1 and CRTC3 antibodies.

To understand the molecular mechanism through which Lkb1 regulates *Ucp1* gene expression, we examined the CRTC3 signalling pathway, which is highly expressed in adipocytes and has important functions in fine-tuning glucose and lipid metabolism, and mitochondrial biogenesis^50^,^51^,^52^. Strikingly, Lkb1 deletion led to apparent nuclear translocation of CRTC3 (Fig. 5a,b), without affecting its total protein levels in adipocytes or adipose tissues (Supplementary Fig. 13a–d). Immunostaining revealed intense CRTC3 signals in the nucleus of Lkb1 KO adipocytes, but not WT adipocytes (Fig. 5a). These results suggest that the higher Ucp1 expression in the *Adipoq-Lkb1* KO mice is correlated to CRTC3 nuclear localization.

To understand how Lkb1 affects the intracellular compartmentalization of CRTC3, we used co-immunoprecipitation (Co-IP) to examine whether these two proteins interact. Indeed, we detected robust pull-down of CRTC3 by Lkb1 antibody in 293T cells after overexpression of these genes and *vice versa* (Fig. 5c–e). Furthermore, we detected endogenous interaction between CRTC3 and Lkb1 proteins in differentiated BAT SVF cells, 3T3-L1 cells and adipose tissues (Supplementary Fig. 14). These data suggest that Lkb1 interacts with CRTC3, and subsequently regulates its intracellular localization.

### CRTC3 binds C/EBPβ to increase its transcriptional activity

We further investigated how nuclear translocation of CRTC3 regulates *Ucp1* gene expression. CRTC3 has been shown to bind to the basic leucine zipper (bZIP) domain of CREB to enhance its transcriptional activity^53^,^54^. As C/EBPβ is also a member of the bZIP transcription factor family^55^ and is essential for brown adipocytes differentiation and Ucp1 expression^56^, we asked whether CRTC3 binds C/EBPβ to regulate BAT gene expression. We first confirmed that overexpression of C/EBPβ in brown adipocytes significantly increased the expression of brown fat related genes including *Ucp1*, *Prdm16* and *Pgc1a* (Supplementary Fig. 15a). In addition, chromatin immunoprecipitation (ChIP) and luciferase reporter assay indicated that C/EBPβ directly binds to the promoter of Ucp1 to enhance *Ucp1* gene transcription (Supplementary Fig. 15b,c).

**Figure 6:**
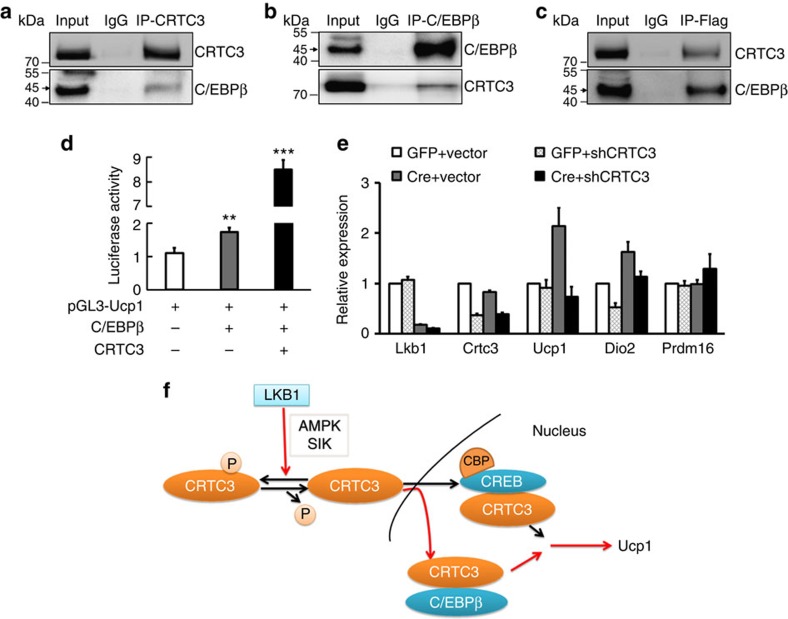
CRTC3 binds to C/EBPβ to enhance its transcriptional activity. (**a**–**c**) CRTC3 interacts with C/EBPβ. 293T cells were co-transfected with pcDNA-Flag-CRTC3 and pcDNA-C/EBPβ, then immunoprecipitated (IP) with CRTC3 (**a**), C/EBPβ (**b**) and Flag (**c**) antibodies, followed by Immunoblotting (IB) with CRTC3 and C/EBPβ antibodies. (**d**) Luciferase assay of 293T cells after co-transfected with the plasmids shown. *n*=4. (**e**) CRTC3 shRNA knockdown abolishes the effects of Lkb1 deletion on Ucp1 expression. BAT preadipocyte from *Lkb1*^*flox/flox*^ mice were infected with adenovirus expressing GFP or Cre, plus adenovirus carrying LacZ or CRTC3 shRNA. After differentiation, total RNA was extracted for Real-time PCR analysis. Error bars represent s.e.m. *n*=4, **P<0.01, *P*<0.001. (**f**) A model depicting Lkb1's role in regulating the localization and function of CRTC3. Red arrows indicate novel pathways shown in this study and black arrows indicate previously published pathways.

We then performed Co-IP experiments after overexpression in 293T cells and found that C/EBPβ can be pulled down by CRTC3 and *vice versa* (Fig. 6a–c). Likewise, endogenous interactions between CRTC3 and C/EBPβ were also found in differentiated BAT SVF cells, 3T3-L1 and BAT (Supplementary Fig. 16), suggesting that CRTC3 physically interacts with C/EBPβ. To examine the function of this interaction, we transfected 293T cells with pcNDA-Flag-CRTC3, pcDNA-C/EBPβ and PGL3–Ucp1 (Ucp1 luciferase reporter) plasmids in a combinatorial fashion. Importantly, co-transfection of CRTC3 markedly increased the transcriptional activity of C/EBPβ on the Ucp1 promoter (Fig. 6d), indicating CRTC3 binds to C/EBPβ and regulates C/EBPβ transcriptional activity.

To directly interrogate whether CRTC3 is necessary to mediate the effects of Lkb1 deletion on Ucp1 expression, we knocked-down CRTC3 in BAT SVF isolated from *Lkb1*^*flox/flox*^ mice. Remarkably, we found that knockdown CRTC3 abolished the induction of *Ucp1* expression by Lkb1 deletion in the subsequently differentiated brown adipocytes (Fig. 6e). Therefore, we conclude that Lkb1 deletion promotes CRTC3 nuclear translocation, where CRTC3 interacts with C/EBPβ to upregulate Ucp1 expression (Fig. 6f).

## Discussion

In this study, we discovered that adipocyte-specific deletion of Lkb1 leads to the expansion of classical BAT and increases the expression of Ucp1 in BAT and sWAT. This subsequently improves glucose metabolism, increases energy expenditure and protects mice from HFD-induced obesity. We delineated that Lkb1 affects the BAT expansion through AMPK-mTOR pathway and provided biochemical evidence that Lkb1 interacts with CRTC3 and deletion of Lkb1 promotes CRTC3 nuclear translocation. Finally, we showed that nuclear CRTC3 binds to C/EBPβ and promotes its transcriptional activity, and Lkb1 deletion upregulates Ucp1 level through CRTC3. It is worth mentioning that although the data reported in some figures (for example, Fig. 2) are derived from male mice, the *Adipoq-Lkb1* KO females exhibit identical phenotype. These results from both genders reveal a critical role of Lkb1 signalling pathway in regulating BAT development and whole-body insulin sensitivity and energy metabolism.

We used a highly adipocyte-specific *Adipoq-Cre* mouse model^42^,^43^ to drive the deletion of Lkb1. Our results are in contrast to the phenotypes observed in *Fabp4-Cre*-driven Lkb1 KO mice (termed *Fabp4-Lkb1* KO henceforth), which develop lipodystrophy in WAT and die prematurely^41^. As *Fabp4-Cre* is expressed in mature adipocytes as well as preadipocytes (adipo-progenitors), the lipodystrophy of WAT in the *Fabp4-Lkb1* KO mice may be due to the key role of Lkb1 in white preadipocyte survival and differentiation. In addition, the leaky expression of *Fabp4-Cre* in non-adipose tissues including endothelial cells^42^,^57^ may have led to off-target effects that contributed to the premature death of the *Fabp4-Lkb1* KO mice. Indeed, it was reported that endothelium-specific deletion of Lkb1 in mice results in premature death^58^,^59^. As white adipocyte differentiation and growth predominantly occur at postnatal stages, the premature death of the *Fabp4-Lkb1* KO mice may have prevented the normal growth of WAT independent of Lkb1 function in adipose *per se*. Surprisingly, *Fabp4-Cre* fails to induce Lkb1 deletion in BAT, even though lineage tracing experiments clearly demonstrate that *Fabp4-Cre* marks a population of progenitors and all mature adipocytes in the BAT^60^. Compared with the *Fabp4-Lkb1* KO, our mouse model not only bypasses the premature lethality but also results in robust deletion of Lkb1 in both WAT and BAT, thus provides an excellent opportunity to unbiasedly assess the role of Lkb1 in adipocytes.

In our indirect calorimetry study, O_2_ consumption and CO_2_ production were normalized by body weight (BW). Other normalization standards in the field include lean mass and per animal^61^. Given that the KO and WT mice have similar BW when fed on chow diet, using BW or per animal for normalization should give the same result. However, the KO mice were lighter than the WT mice after HFD feeding, mainly due to changes in fat and liver mass, we choose to normalize the values to BW to reflect such changes. Recently, Tschoep *et al.*^61^ suggested that combining effects of different tissues and using analysis of covariance would facilitate unbiased comparison of energy expenditure measurements across studies.

It is interesting that *Adipoq-Lkb1* KO BAT on one hand appears to be morphologically whitened but on the other hand expresses higher levels of Ucp1 and exhibits higher thermogenic activities (based on OCR analysis). This paradoxical observation suggests that thermogenic activity of adipocytes is not always associated with the size and number of lipid droplets. In the Lkb1 KO mice, hypertrophy of brown adipocytes may have simultaneously increased their lipid storage (size of lipid droplets) and thermogenic capacities. Supporting this idea, both white and brown adipocytes are hypertrophic (that is, contain larger lipid droplets) in the *Adipoq-Pten* KO mice but the mice have enhanced insulin sensitivity^62^. Alternatively, the increased lipid storage in *Adipoq-Lkb1* KO brown adipocytes may be an adaptive mechanism to allow sufficient lipid supply to match a greater rate of lipid utilization. It is well-known that lipolysis of triacylglycerol (lipid droplets) generates free fatty acids that subsequently activates Ucp1 (ref. 63). Other groups have reported that browning conditions (such as β3-adrenergic receptor activation and cold exposure) elevate fatty acid synthase (FAS) in BAT to promote free fatty acids re-esterification and triacylglycerol formation^64^,^65^. Thus, concomitant increases in lipid storage and utilization may explain the BAT phenotypes in the *Adipoq-Lkb1* KO mice.

Loss function of Lkb1 in WAT and BAT cells promoted adipocyte differentiation and lipid accumulation, accompanied by increased expression of brown/beige specific genes. Conversely, Lkb1 overexpression reduced the lipid accumulation, and suppressed expression of brown/beige-specific genes. However, we noted that the extent of changes in differentiation markers was lesser than the extent of changes in BAT-specific gene expression. Therefore, changes in BAT marker gene expression may be partially due to alterations in overall differentiation.

AMPK is a canonical substrate of Lkb1 and an energy sensor through interaction with mTOR signalling pathway^66^. We found that deletion of Lkb1 decreases the activity and phosphorylation of AMPK while increasing the phosphorylation of S6, an established downstream target of mTOR. This result suggests that Lkb1 regulates mTOR pathway through AMPK in adipocytes. As mTOR pathway plays positive roles in regulating the fat mass and fat cell size^48^,^49^,^67^, augmentation of mTOR signalling in the *Adipoq-Lkb1* KO mice explains the BAT expansion phenotype. Double deletion of Lkb1 and mTOR provides the definitive proof that Lkb1 regulates BAT mass through mTOR.

We discovered a novel interaction between Lkb1 and CRTC3 that controls the intracellular localization of CRTC3, a transcriptional co-activator that is highly expressed in adipose tissues^51^. The CRTC family proteins possess a highly conserved N-terminal CREB-binding domain, which interacts with the bZIP domain of CREB and to regulate its activity^53^,^68^,^69^,^70^. Under basal conditions, CRTC1 and CRTC2 are mainly sequestered in the cytoplasm through phosphorylation and interactions with 14-3-3 proteins^51^,^54^, while CRTC3 is thought to be mainly localized in the nucleus^70^. However, we found that CRTC3 was mainly localized in cytoplasm of brown adipocytes in the WT mice, and Lkb1 deletion induced nuclear translocation of CRTC3. Dephosphorylation-mediated nuclear translocation of CRTCs is a critical and conserved step governing their function in upregulating CREB target genes^53^,^54^. Previous studies have shown that Lkb1 substrates such as AMPK and SIK can phosphorylate CRTCs and inhibit CREB-mediated gene expression^70^,^71^,^72^. As such, CRTC2 is a critical downstream target of LKB1/AMPK signals in the regulation of liver gluconeogenesis^34^. Here we found that Lkb1 directly interacts with CRTC3. We speculate that the protein kinase domain of Lkb1 interacts with the central regulatory region of CRTC3, where Lkb1 phosphorylates the Ser residue of CRTC3 to regulate its translocation between cytoplasm and nuclear. On the basis of a previous study^73^ and online protein software (Uniport) analysis, phosphorylation of Ser62, Ser162, Ser329, Ser370, Ser391 and Ser443 may be involved in the intracellular localization of CRTC3. However, pinpointing the exact Lkb1 phosphorylation site requires further biochemical analysis.

We demonstrate in this study that CRTC3 interacts with C/EBPβ to promote its transcriptional activity. C/EBPβ has been reported to play an important role in regulating BAT differentiation and gene expression^56^. Consistently, we found that overexpression C/EBPβ in BAT cells potently induces expression of BAT-related genes. ChIP and luciferase assays further showed that C/EBPβ directly binds to Ucp1 promoter to upregulate its expression. Importantly, luciferase assay indicates that CRTC3 upregulates the transcriptional activity of C/EBPβ on the Ucp1 promoter, thus demonstrating the functional significance of the CRTC3-C/EBPβ interaction.

Our conclusion that Lkb1 KO upregulates *Ucp1* expression through CRTC3 is supported by knockdown assays in which knocking down CRTC3 in Lkb1 null adipocytes abolishes *Ucp1* upregulation. Likewise, in muscle and liver cells, CRTC3 markedly induce PGC-1 (an upstream regulator of Ucp1) transcription and mitochondrial biogenesis^74^,^75^. Interestingly, previous reports have shown that whole-body KO of CRTC3 increases energy expenditure and protect the mutant mice from obesity and hepatic steatosis^51^, which appear to contradict our observation that CTRC3 knockdown prevents Ucp1 upregulation in Lkb1 KO adipocytes. However, the previous study is inconsistent to the overall phenotype of the *Adipoq-Lkb1* KO mice in which the total protein levels of CRTC3 were not affected. Instead, what seems to be most important in adipocytes is the translocation of CRTC3 into the nucleus, where it acts as a novel co-activator of C/EBPβ to regulate *Ucp1* gene expression.

## Methods

### Animals

All procedures involving mice were approved by Purdue University Animal Care and Use Committee. The *Adipoq-Cre* (stock #010803), *Lkb1*^*flox/flox*^ (stock #014143) and *mTOR*^*flox/flox*^ (stock #011009) mice were purchased from Jackson Laboratory (Bar Harbor, ME). Mice were in a C57BL/6J background and housed in the animal facility with free access to water and standard rodent chow food or HFD (TD.06414 Harlan). PCR genotyping was done using protocols described by the supplier. Food intake assay was calculated by measuring food consumption weekly. Unless otherwise indicated, 2–5 months old adult mice of both sexes were analysed in this study.

### Indirect calorimetry study

Oxygen consumption (VO_2_), carbon dioxide production (VCO_2_), respiratory exchange ratios and heat production were measured by using indirect calorimetry system (Oxymax, Columbus Instruments), installed under a constant environmental temperature (22 or 30 °C) and a 12-h light (06:00–18:00 hours), 12-h dark cycle (18:00–06:00 hours). Mice in each chamber had free access to food and water. The raw data were normalized by body weight and the histograms of day (06:00–18:00 hours) and night (18:00–06:00 hours) values were the mean value of all points measured during the 12-h period.

### Blood glucose measurements

For GTT, mice were given i.p. injection of 100 mg ml^−1^ D-glucose (2 g kg^−1^ body weight) after overnight fasting, and tail blood glucose concentrations were measured by a glucometer (Accu-Check Active, Roche). For ITT, mice were fasted for 4 h before i.p. administration of human insulin (Santa Cruz) (0.75 U per kg body weight), and tail blood glucose concentrations were monitored. For both GTT and ITT, each mouse was singly caged with blinded cage number and random orders.

### H&E staining and immunostaining

Adipose tissues from the WT and *Adipoq-Lkb1* mice were fixed in 10% formalin for 24 h at room temperature. Then the tissues were embedded into paraffin, blocked and cut at 3–10 μm for H&E staining and immunostaining. For H&E staining, the sections were deparaffinized, rehydrated and the nuclei stained with haematoxylin for 15 min. Sections were then rinsed in running tap water and stained with eosin for 1 min, dehydrated and mounted. Whole-slide digital images were collected at × 20 magnification with an Aperio Scan Scope slide scanner (Aperio, Vista, CA). Scanned images of H&E staining were analysed by Photoshop CS3 to calculate nuclei numbers. For immunostaining, the sections were blocked with blocking buffer containing 5% goat serum, 2% BSA, 0.2% triton X-100 and 0.1% sodium azide in PBS for 1 h after deparaffinized and antigen retrieval. Then the samples were incubated with CRTC3 (Cell Signalling, C35G4) and Ki67 (Abcam, ab-16667) primary antibodies diluted in blocking buffer overnight. After washing with PBS, the samples were incubated with secondary antibodies and DAPI for 45 min at room temperature. Fluorescent images were captured as single-channel grayscale images using a Leica DM 6000B fluorescent microscope with a × 20 objective (NA 0.70). Images for WT and conditional KO samples were captured using identical parameters and both WT and mutant images were adjusted identically in Photoshop.

### Cell culture

Primary BAT and WAT SVF cells were isolated using collagenase digestion and followed by density separation. Briefly, the interscapular brown adipose and inguinal white adipose were minced and digested in 1.5 mg ml^−1^ collagenase at 37 °C for 0.5 and 1 h, respectively. The digestions were terminated with DMEM containing 10% FBS, and filtered through 100-μm filters to remove connective tissues and undigested trunks of tissues. Cells were then centrifuged at 450*g* for 5 min to separate the SVF cells in the sediment and lipid-containing adipocytes in the floating layer. The freshly isolated SVF cells were seeded and cultured in growth medium containing DMEM, 20% FBS, 1% penicillin/streptomycin (P/S) at 37 °C with 5% CO_2_ for 3 days, followed by feeding with fresh medium every 2 days. The BAT cell line (kindly provided by Professor Yongxu Wang, University of Massachusetts Medical School), were cultured in same condition as SVF cells, while 3T3-L1 (ATCC) and HEK293T (ATCC) were cultured in DMEM with 10% FBS. For SVF cell and BAT cell line adipogenic differentiation, the cells were induced with induction medium contains DMEM, 10% FBS, 2.85 μM insulin, 0.3 μM dexamethasone, 1 μM rosiglitazone, and 0.63 mM 3-isobutyl-methylxanthine for 3 days on confluence and then differentiated in differentiation medium contains DMEM, 10% FBS, 200 nM insulin and 10 nM T3 for 4 days until adipocytes mature. To avoid the effect of cell density on adipogenic differentiation, cells were induced to differentiate when they reach 90% confluence. For 3T3-L1 adipogenic differentiation, cells of 100% confluence were kept in growth medium for 2 days then induced with induction medium for 2 days, after that differentiated in differentiation medium (without T3) for 6 days. Mycoplasma was certified by ATCC when cells were purchased. All cell lines were periodically tested for identity using PCR and by morphological features.

### Oil red O staining

Cultured cells were washed with PBS and fixed with 10% formaldehyde for 15 min at room temperature. Then the cells were stained using the Oil red O working solutions containing 6 ml Oil red O stock solution (5 g l^−1^ in isopropanol) and 4 ml ddH_2_O for 30 min. After staining, the cells were washed with 60% isopropanol and pictured. Triglycerides were extracted from Oil Red O-stained adipocytes with 100% isopropanol, and the triglyceride contents were analysed by measuring the optical density at 490 nm (OD 490).

### Total RNA extraction and real-time PCR

Total RNA was extracted from cells or tissues using Trizol Reagent according to the manufacturer's instructions. RNA was treated with RNase-free DNase l to remove contaminating genomic DNA. The purity and concentration of total RNA were measured by a spectrophotometer (Nanodrop 3000, Thermo Fisher) at 260 and 280 nm. Ratios of absorption (260/280 nm) of all samples were between 1.8 and 2.0. Then 5 μg of total RNA were reversed transcribed using random primers and MMLV reverse transcriptase. Real-time PCR was carried out with a Roche Lightcycler 480 PCR System using SYBR Green Master Mix and gene-specific primers. Primer sequences are listed in Supplementary Table 1. The 2^−ΔΔCT^ method was used to analyse the relative changes in gene expression normalized against 18S ribosomal RNA as internal control.

### Protein extraction and western blot analysis

Total protein was isolated from cells or tissues using RIPA buffer contains 50 mM Tris-HCl (pH 8.0), 150 mM NaCl, 1% NP-40, 0.5% sodium Deoxycholate and 0.1% SDS. The cytoplasm fractions were extract with buffer A contains 10 mM HEPES (pH 7.4), 1 mM KCl, 1 mM EDTA, 1Mm EGTA, 1 mM dithiothreitol, 0.5% Nonidet P-40 and protease inhibitor. After extracted the cytoplasm proteins, the nuclei pellet was washed with buffer A (without Nonidet P-40) and resolved by RIPA buffer for nuclear proteins. Protein concentrations were determined using Pierce BCA Protein Assay Reagent (Pierce Biotechnology). Proteins were separated by SDS–PAGE, transferred to a polyvinylidene fluoride membrane (Millipore Corporation), blocking in 5% fat-free milk for 1 h at RT, then incubated with first antibodies in 5% milk overnight at 4 °C. The CRTC3 antibody was from Cell Signalling (C35G4, 1:1,000), Ucp1 was from Abcam (ab23841, 1:2,000), all other antibodies were from Santa Cruz Biotechnology (Santa Cruz), including Lkb1 (sc-32245, 1:1,000), phosphor-AMPK (sc-33524, 1:1,000), AMPK (sc-25792, 1: 1,000), C/EBPβ (sc-150, 1:1,000), SIK (sc-83754, 1:1,000) and GAPDH (sc-32233, 1:1,000). The horseradish peroxidase (HRP)-conjugated secondary antibody (anti-rabbit IgG, 111-035-003 or anti-mouse IgG; 115-035-003, Jackson ImmunoResearch) or infrared secondary antibody (Alexa Fluor 790 goat anti-mouse IgG, A11357Life Technologies, USA) were diluted 1:5,000 and 1:10,000, respectively. Immunodetection was performed using enhanced chemiluminescence western blotting substrate (Pierce Biotechnology) and detected with FluorChem R System (ProteinSimple). Results shown in the figures are representative results from at least three independent experiments. Uncropped original gel images are available in Supplementary Fig. 17.

### ChIP assay

Brown fat from the mutant mice were dissected, washed with PBS, minced and fixed with 1% formaldehyde in DMEM for 10 min at room temperature followed by the addition of 125 mM glycine for 5 min at room temperature, after which samples were washed twice with cold PBS and placed in SDS lysis buffer containing 20 mM Tris, 0.1% SDS, 1% Triton-100, 150 mM NaCl, 1 Mm EDTA and protease inhibitor. The samples were further sonicated and diluted for IP with the indicated antibodies (anti-C/EBPβ, 1:50) and incubation at 4 °C overnight. Then, the immunoprecipitates were eluted and reverse crosslinked overnight at 65 °C. DNA fragments were purified using the Cycle Pure kit (Omega Bio-Tek), and quantitative PCR was performed.

### Luciferase assay

HEK293T cells were seeded into 48-well plates 1 day before Lipofectamine 2000–mediated transfection. The pGL3–Ucp1 promoter luciferase plasmid was generated. For transfection of each well, 80 ng Renilla plasmid, 250 ng pGL3–Ucp1 and 500 ng pcDNA-C/EBPβ plasmid (or its blank control plasmid) and/or 500 ng pcDNA-flag-CRTC3 (or its blank control plasmid) were co-transfected following the manufacturer's instructions. Cells were harvested 36 h after transfection and analysed with the Dual-Luciferase Reporter Assay System (Promega).

### Co-IP assay

Total protein was extracted from HEK293T cells, transfected with pcDNA-flag-CRTC3 or/and pcDNA-C/EBPβ plasmid after 36 h, differentiated 3T3-L1, BAT cell line and BAT. The lysate was precleared with protein A/G agarose at 4 °C for 1 h. Then 2 μg of primary antibody anti-CRTC3, anti-Lkb1, anti-CEBPβ or anti-Flag was added into lysate contains 500 μg total protein and rotating at 4 °C overnight. The next morning added the protein A/G agarose and rotating for 2 h. The samples were washed with cold PBS for three times and collected for western blot.

### Adipocyte OCR measurement

Primary SVF cells from BAT and inguinal WAT were isolated and cultured for 3 days before being plated in XF cell culture microplates (Seahorse Bioscience). SVF cells (10,000 cells) were seeded in each well, and each treatment included cells from seven BAT or three WAT replicates. After differentiated 6 d, cultured adipocytes were washed twice and pre-incubated in XF medium (supplemented with 25 mM glucose, 2 mM glutamine and 1 mM pyruvate) for 1–2 h at 37 °C without CO_2_. The OCR was measured using the XF Extracellular Flux Analyser (Seahorse Biosciences). Oligomycin (2 mM), FCCP (2 mM), and Antimycin A and Rotenone (0.5 μM) were preloaded into cartridges and injected into XF wells in succession. OCR was calculated as a function of time (picomoles per minute).

### *In vitro* kinase assay

IP purified AMPK and SIK from BAT lysates (each sample with the same amount of total protein) was, respectively, incubated with AMARA substrate peptide (Abcam, ab204852), bound on phosphocellulose paper in kinase reaction buffer (50 mM Tris, pH 7.5, 10 mM MgCl2, 2 mM EGTA, 0.5 mM Na3VO4, 100 mM 4-nitrophenyl phosphate di(tris) salt (PNPP), 25 mM dithiothreitol (DTT), 125 μM ATP) supplemented with 10 μCi of [^32^P] ATP at 30 °C for 30 min. Then the paper was washed, dried and detected by autoradiography.

### Data analysis

Trial experiments or experiments done previously were used to determine sample size with adequate statistical power. Measurement values that were beyond the boundary determined by the interquartile range were considered as outliers and were excluded from statistical analyses. Specifically, all data from one WT control mouse were excluded from data analysis in Supplementary Fig. 11 due to blood glucose level at one time point was above the detection range of the glucometer used. No other data were excluded and data are presented as means±s.e.m. Comparisons were made by two-tailed Student's *t*-tests. Effects were considered significant at *P*<0.05. The researchers involved in the study were not completely blinded during sample collection or data analysis.

### Data availability

The data that support the findings of this study are available from the corresponding author on request.

## Additional information

**How to cite this article:** Shan, T. *et al.* Lkb1 controls brown adipose tissue growth and thermogenesis by regulating the intracellular localization of CRTC3. *Nat. Commun.* 7:12205 doi: 10.1038/ncomms12205 (2016).

## Supplementary Material

Supplementary Information Supplementary Figure 1-17 and Supplementary Table 1

Supplementary Movie 1 Movement of WT (left) and Adipoq-Lkb1 (right) mice in the new cages.

## References

[b1] Cohen P. *et al.* Ablation of PRDM16 and beige adipose causes metabolic dysfunction and a subcutaneous to visceral fat switch. Cell 156, 304–316 (2014). 24439384 10.1016/j.cell.2013.12.021 PMC3922400

[b2] Wu J., Cohen P. & Spiegelman B. M. Adaptive thermogenesis in adipocytes: is beige the new brown? Genes Dev. 27, 234–250 (2013). 23388824 10.1101/gad.211649.112 PMC3576510

[b3] Nedergaard J. & Cannon B. The changed metabolic world with human brown adipose tissue: therapeutic visions. Cell Metab. 11, 268–272 (2010). 20374959 10.1016/j.cmet.2010.03.007

[b4] Rosen E. D. & Spiegelman B. M. Adipocytes as regulators of energy balance and glucose homeostasis. Nature 444, 847–853 (2006). 17167472 10.1038/nature05483 PMC3212857

[b5] Bi P. *et al.* Inhibition of Notch signalling promotes browning of white adipose tissue and ameliorates obesity. Nat. Med. 20, 911–918 (2014). 25038826 10.1038/nm.3615 PMC4181850

[b6] Bostrom P. *et al.* A PGC1-alpha-dependent myokine that drives brown-fat-like development of white fat and thermogenesis. Nature 481, 463–468 (2012). 22237023 10.1038/nature10777 PMC3522098

[b7] Cao L. *et al.* White to brown fat phenotypic switch induced by genetic and environmental activation of a hypothalamic-adipocyte axis. Cell Metab. 14, 324–338 (2011). 21907139 10.1016/j.cmet.2011.06.020 PMC3172615

[b8] Ohno H., Shinoda K., Spiegelman B. M. & Kajimura S. PPARgamma agonists induce a white-to-brown fat conversion through stabilization of PRDM16 protein. Cell Metab. 15, 395–404 (2012). 22405074 10.1016/j.cmet.2012.01.019 PMC3410936

[b9] Kusminski C. M., Park J. & Scherer P. E. MitoNEET-mediated effects on browning of white adipose tissue. Nat. Commun. 5, 3962 (2014). 24865177 10.1038/ncomms4962 PMC4084619

[b10] Geurts L. *et al.* Adipose tissue NAPE-PLD controls fat mass development by altering the browning process and gut microbiota. Nat. Commun. 6, 6495 (2015). 25757720 10.1038/ncomms7495 PMC4382707

[b11] Rosenwald M., Perdikari A., Rulicke T. & Wolfrum C. Bi-directional interconversion of brite and white adipocytes. Nat. Cell Biol. 15, 659–667 (2013). 23624403 10.1038/ncb2740

[b12] Lee Y. K. & Cowan C. A. White to brite adipocyte transition and back again. Nat. Cell Biol. 15, 568–569 (2013). 23728463 10.1038/ncb2776

[b13] Wang J. *et al.* Ablation of LGR4 promotes energy expenditure by driving white-to-brown fat switch. Nat. Cell Biol. 15, 1455–1463 (2013). 24212090 10.1038/ncb2867

[b14] Moisan A. *et al.* White-to-brown metabolic conversion of human adipocytes by JAK inhibition. Nat. Cell Biol. 17, 57–67 (2015). 25487280 10.1038/ncb3075 PMC4276482

[b15] Seale P. *et al.* Prdm16 determines the thermogenic program of subcutaneous white adipose tissue in mice. J. Clin. Invest. 121, 96–105 (2011). 21123942 10.1172/JCI44271 PMC3007155

[b16] Dempersmier J. *et al.* Cold-inducible Zfp516 activates UCP1 transcription to promote browning of white fat and development of brown fat. Mol. Cell 57, 235–246 (2015). 25578880 10.1016/j.molcel.2014.12.005 PMC4304950

[b17] Crane J. D. *et al.* Inhibiting peripheral serotonin synthesis reduces obesity and metabolic dysfunction by promoting brown adipose tissue thermogenesis. Nat. Med. 21, 166–172 (2015). 25485911 10.1038/nm.3766 PMC5647161

[b18] Rosen E. D. & Spiegelman B. M. What we talk about when we talk about fat. Cell 156, 20–44 (2014). 24439368 10.1016/j.cell.2013.12.012 PMC3934003

[b19] Stanford K. I. *et al.* Brown adipose tissue regulates glucose homeostasis and insulin sensitivity. J. Clin. Invest. 123, 215–223 (2013). 23221344 10.1172/JCI62308 PMC3533266

[b20] Lowell B. B. *et al.* Development of obesity in transgenic mice after genetic ablation of brown adipose-tissue. Nature 366, 740–742 (1993). 8264795 10.1038/366740a0

[b21] Cypess A. M. *et al.* Activation of human brown adipose tissue by a beta 3-adrenergic receptor agonist. Cell Metab. 21, 33–38 (2015). 25565203 10.1016/j.cmet.2014.12.009 PMC4298351

[b22] Shinoda K. *et al.* Genetic and functional characterization of clonally derived adult human brown adipocytes. Nat. Med. 21, 389–394 (2015). 25774848 10.1038/nm.3819 PMC4427356

[b23] Jespersen N. Z. *et al.* A classical brown adipose tissue mRNA signature partly overlaps with brite in the supraclavicular region of adult humans. Cell Metab. 17, 798–805 (2013). 23663743 10.1016/j.cmet.2013.04.011

[b24] Cypess A. M. *et al.* Identification and importance of brown adipose tissue in adult humans. N. Engl. J. Med. 360, 1509–1517 (2009). 19357406 10.1056/NEJMoa0810780 PMC2859951

[b25] van Marken Lichtenbelt W. D. *et al.* Cold-activated brown adipose tissue in healthy men. N. Engl. J. Med. 360, 1500–1508 (2009). 19357405 10.1056/NEJMoa0808718

[b26] Virtanen K. A. *et al.* Functional brown adipose tissue in healthy adults. N. Engl. J. Med. 360, 1518–1525 (2009). 19357407 10.1056/NEJMoa0808949

[b27] Lidell M. E. *et al.* Evidence for two types of brown adipose tissue in humans. Nat. Med. 19, 631–634 (2013). 23603813 10.1038/nm.3017

[b28] Yoneshiro T. *et al.* Recruited brown adipose tissue as an antiobesity agent in humans. J. Clin. Invest. 123, 3404–3408 (2013). 23867622 10.1172/JCI67803 PMC3726164

[b29] Hemminki A. *et al.* A serine/threonine kinase gene defective in Peutz-Jegheus syndrome. Nature 391, 184–187 (1998). 9428765 10.1038/34432

[b30] Jenne D. E. *et al.* Peutz-Jeghers syndrome is caused by mutations in a novel serine threonine kinase. Nat. Genet. 18, 38–44 (1998). 9425897 10.1038/ng0198-38

[b31] Nakada D., Saunders T. L. & Morrison S. J. Lkb1 regulates cell cycle and energy metabolism in haematopoietic stem cells. Nature 468, 701–704 (2010). 21124450 10.1038/nature09571 PMC3059717

[b32] Granot Z. *et al.* LKB1 Regulates Pancreatic beta Cell Size, Polarity, and Function. Cell Metab. 10, 296–308 (2009). 19808022 10.1016/j.cmet.2009.08.010 PMC2790403

[b33] Ji H. *et al.* LKB1 modulates lung cancer differentiation and metastasis. Nature 448, 807–810 (2007). 17676035 10.1038/nature06030

[b34] Shaw R. J. *et al.* The kinase LKB1 mediates glucose homeostasis in liver and therapeutic effects of metformin. Science 310, 1642–1646 (2005). 16308421 10.1126/science.1120781 PMC3074427

[b35] Fu A. *et al.* Loss of Lkb1 in adult beta cells increases beta cell mass and enhances glucose tolerance in mice. Cell Metab. 10, 285–295 (2009). 19808021 10.1016/j.cmet.2009.08.008

[b36] Ikeda Y. *et al.* Cardiac-specific deletion of LKB1 leads to hypertrophy and dysfunction. J. Biol. Chem. 284, 35839–35849 (2009). 19828446 10.1074/jbc.M109.057273 PMC2791013

[b37] Jeppesen J. *et al.* LKB1 regulates lipid oxidation during exercise independently of AMPK. Diabetes 62, 1490–1499 (2013). 23349504 10.2337/db12-1160 PMC3636614

[b38] Koh H. J. *et al.* Skeletal muscle-selective knockout of LKB1 increases insulin sensitivity, improves, glucose homeostasis, and decreases TRB3. Mol. Cell. Biol. 26, 8217–8227 (2006). 16966378 10.1128/MCB.00979-06 PMC1636784

[b39] Sakamoto K. *et al.* Deficiency of LKB1 in skeletal muscle prevents AMPK activation and glucose uptake during contraction. EMBO J. 24, 1810–1820 (2005). 15889149 10.1038/sj.emboj.7600667 PMC1142598

[b40] Shan T. *et al.* Lkb1 is indispensable for skeletal muscle development, regeneration, and satellite cell homeostasis. Stem Cells 32, 2893–2907 (2014). 25069613 10.1002/stem.1788 PMC4198532

[b41] Zhang W., Wang Q., Song P. & Zou M. H. Liver kinase b1 is required for white adipose tissue growth and differentiation. Diabetes 62, 2347–2358 (2013). 23396401 10.2337/db12-1229 PMC3712073

[b42] Lee K. Y. *et al.* Lessons on conditional gene targeting in mouse adipose tissue. Diabetes 62, 864–874 (2013). 23321074 10.2337/db12-1089 PMC3581196

[b43] Eguchi J. *et al.* Transcriptional control of adipose lipid handling by IRF4. Cell Metab. 13, 249–259 (2011). 21356515 10.1016/j.cmet.2011.02.005 PMC3063358

[b44] Orava J. *et al.* Different metabolic responses of human brown adipose tissue to activation by cold and insulin. Cell Metab. 14, 272–279 (2011). 21803297 10.1016/j.cmet.2011.06.012

[b45] Nedergaard J. & Cannon B. The Browning of White Adipose Tissue: Some Burning Issues. Cell Metab. 20, 396–407 (2014). 25127354 10.1016/j.cmet.2014.07.005

[b46] Katoh Y. *et al.* Salt-inducible kinase (SIK) isoforms: their involvement in steroidogenesis and adipogenesis. Mol. Cell. Endocrinol. 217, 109–112 (2004). 15134808 10.1016/j.mce.2003.10.016

[b47] Okamoto M., Takemori H. & Katoh Y. Salt-inducible kinase in steroidogenesis and adipogenesis. Trends Endocrinol. Metab. 15, 21–26 (2004). 14693422 10.1016/j.tem.2003.11.002

[b48] Polak P. *et al.* Adipose-specific knockout of raptor results in lean mice with enhanced mitochondrial respiration. Cell Metab. 8, 399–410 (2008). 19046571 10.1016/j.cmet.2008.09.003

[b49] Um S. H. *et al.* Absence of S6K1 protects against age- and diet-induced obesity while enhancing insulin sensitivity. Nature 431, 200–205 (2004). 15306821 10.1038/nature02866

[b50] Qi L. *et al.* Adipocyte CREB promotes insulin resistance in obesity. Cell Metab. 9, 277–286 (2009). 19254572 10.1016/j.cmet.2009.01.006 PMC2730923

[b51] Song Y. *et al.* CRTC3 links catecholamine signalling to energy balance. Nature 468, 933–U329 (2010). 21164481 10.1038/nature09564 PMC3025711

[b52] Wang Y. G., Vera L., Fischer W. H. & Montminy M. The CREB coactivator CRTC2 links hepatic ER stress and fasting gluconeogenesis. Nature 460, 534–537 (2009). 19543265 10.1038/nature08111 PMC2730924

[b53] Bittinger M. A. *et al.* Activation of cAMP response element-mediated gene expression by regulated nuclear transport of TORC proteins. Curr. Biol. 14, 2156–2161 (2004). 15589160 10.1016/j.cub.2004.11.002

[b54] Altarejos J. Y. & Montminy M. CREB and the CRTC co-activators: sensors for hormonal and metabolic signals. Nat. Rev. Mol. Cell. Biol. 12, 141–151 (2011). 21346730 10.1038/nrm3072 PMC4324555

[b55] Hu H. M. *et al.* The C/EBP bZIP domain can mediate lipopolysaccharide induction of the proinflammatory cytokines interleukin-6 and monocyte chemoattractant protein-1. J. Biol. Chem. 275, 16373–16381 (2000). 10748205 10.1074/jbc.M910269199

[b56] Tanaka T., Yoshida N., Kishimoto T. & Akira S. Defective adipocyte differentiation in mice lacking the C/EBPbeta and/or C/EBPdelta gene. EMBO J. 16, 7432–7443 (1997). 9405372 10.1093/emboj/16.24.7432 PMC1170343

[b57] Urs S., Harrington A., Liaw L. & Small D. Selective expression of an aP2/fatty acid binding protein4-Cre transgene in non-adipogenic tissues during embryonic development. Transgenic. Res. 15, 647–653 (2006). 16952017 10.1007/s11248-006-9000-z

[b58] Londesborough A. *et al.* LKB1 in endothelial cells is required for angiogenesis and TGF beta-mediated vascular smooth muscle cell recruitment. Development 135, 2331–2338 (2008). 18539926 10.1242/dev.017038

[b59] Zhang W. C. *et al.* Endothelial cell-specific liver kinase B1 deletion causes endothelial dysfunction and hypertension in mice *in vivo*. Circulation 129, 1428–1439 (2014). 24637557 10.1161/CIRCULATIONAHA.113.004146 PMC3972325

[b60] Shan T., Liu W. & Kuang S. Fatty acid binding protein 4 expression marks a population of adipocyte progenitors in white and brown adipose tissues. FASEB J. 27, 277–287 (2013). 23047894 10.1096/fj.12-211516 PMC3528316

[b61] Tschop M. H. *et al.* A guide to analysis of mouse energy metabolism. Nat. Methods 9, 57–63 (2012). 22205519 10.1038/nmeth.1806 PMC3654855

[b62] Morley T. S., Xia J. Y. & Scherer P. E. Selective enhancement of insulin sensitivity in the mature adipocyte is sufficient for systemic metabolic improvements. Nat. Commun. 6, 7906 (2015). 26243466 10.1038/ncomms8906 PMC4527086

[b63] Nicholls D. G. & Rial E. A history of the first uncoupling protein, UCP1. J. Bioenerg. Biomembr. 31, 399–406 (1999). 10653469 10.1023/a:1005436121005

[b64] Mottillo E. P. *et al.* Coupling of lipolysis and *de novo* lipogenesis in brown, beige, and white adipose tissues during chronic beta3-adrenergic receptor activation. J. Lipid Res. 55, 2276–2286 (2014). 25193997 10.1194/jlr.M050005 PMC4617130

[b65] Yu X. X., Lewin D. A., Forrest W. & Adams S. H. Cold elicits the simultaneous induction of fatty acid synthesis and beta-oxidation in murine brown adipose tissue: prediction from differential gene expression and confirmation *in vivo*. FASEB J. 16, 155–168 (2002). 11818363 10.1096/fj.01-0568com

[b66] Lizcano J. M. *et al.* LKB1 is a master kinase that activates 13 kinases of the AMPK subfamily, including MARK/PAR-1. EMBO J. 23, 833–843 (2004). 14976552 10.1038/sj.emboj.7600110 PMC381014

[b67] Murakami M. *et al.* mTOR is essential for growth and proliferation in early mouse embryos and embryonic stem cells. Mol. Cell. Biol. 24, 6710–6718 (2004). 15254238 10.1128/MCB.24.15.6710-6718.2004 PMC444840

[b68] Iourgenko V. *et al.* Identification of a family of cAMP response element-binding protein coactivators by genome-scale functional analysis in mammalian cells. Proc. Natl Acad. Sci. USA 100, 12147–12152 (2003). 14506290 10.1073/pnas.1932773100 PMC218727

[b69] Conkright M. D. *et al.* TORCs: Transducers of regulated CREB activity. Mol. Cell 12, 413–423 (2003). 14536081 10.1016/j.molcel.2003.08.013

[b70] Screaton R. A. *et al.* The CREB coactivator TORC2 functions as a calcium- and cAMP-sensitive coincidence detector. Cell 119, 61–74 (2004). 15454081 10.1016/j.cell.2004.09.015

[b71] Koo S. H. *et al.* The CREB coactivator TORC2 is a key regulator of fasting glucose metabolism. Nature 437, 1109–1111 (2005). 16148943 10.1038/nature03967

[b72] Mair W. *et al.* Lifespan extension induced by AMPK and calcineurin is mediated by CRTC-1 and CREB. Nature 470, 404–408 (2011). 21331044 10.1038/nature09706 PMC3098900

[b73] Clark K. *et al.* Phosphorylation of CRTC3 by the salt-inducible kinases controls the interconversion of classically activated and regulatory macrophages. Proc. Natl Acad. Sci. USA 109, 16986–16991 (2012). 23033494 10.1073/pnas.1215450109 PMC3479463

[b74] Wu Z. *et al.* Transducer of regulated CREB-binding proteins (TORCs) induce PGC-1alpha transcription and mitochondrial biogenesis in muscle cells. Proc. Natl Acad. Sci. USA 103, 14379–14384 (2006). 16980408 10.1073/pnas.0606714103 PMC1569674

[b75] Than T. A., Lou H., Ji C., Win S. & Kaplowitz N. Role of cAMP-responsive element-binding protein (CREB)-regulated transcription coactivator 3 (CRTC3) in the initiation of mitochondrial biogenesis and stress response in liver celle. J. Biol. Chem. 286, 22047–22054 (2011). 21536665 10.1074/jbc.M111.240481 PMC3121349

