# Supplementary material for: Lkb1 controls brown adipose tissue growth and thermogenesis by regulating the intracellular localization of CRTC3
Source: Nat Commun. 2016 Jul 27;7:12205. doi: 10.1038/ncomms12205 (PMC4974456; doi:10.1038/ncomms12205)
Supplement: Supplementary Information — Supplementary Figure 1-17 and Supplementary Table 1 [file ncomms12205-s1.pdf]

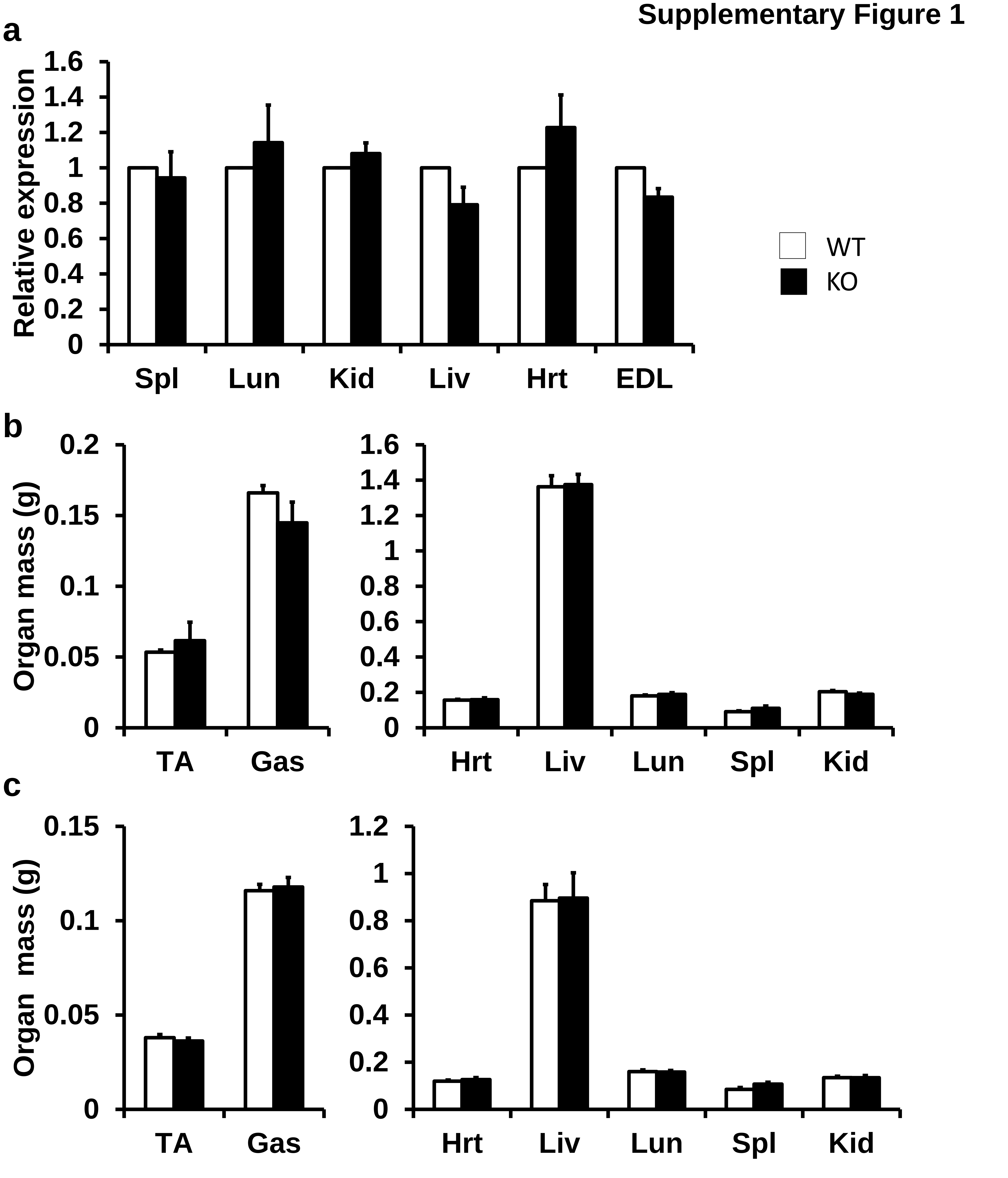

Supplementary Figure 1. Adipose-specific knockout (KO) of Lkb1does not affect Lkb1 expression or mass of non-adipose tissues. (**a**) Relative levels of Lkb1 mRNA in several non-adipose tissues (n=4). (**b, c**) Masses of other organs in WT and KO mice. (**b**) Male mice (WT n=11, KO n=8, 10-wk-old); (**c**) Female mice (WT n=13, KO n=9, 9-wk-old). Error bars: s.e.m. TA, tibialis anterior muscle; Gas, gastrocnemius muscle; Hrt, heart; Liv, liver; Lun, lung; Spl, spleen; Kid, kidney.

Supplementary Figure 2

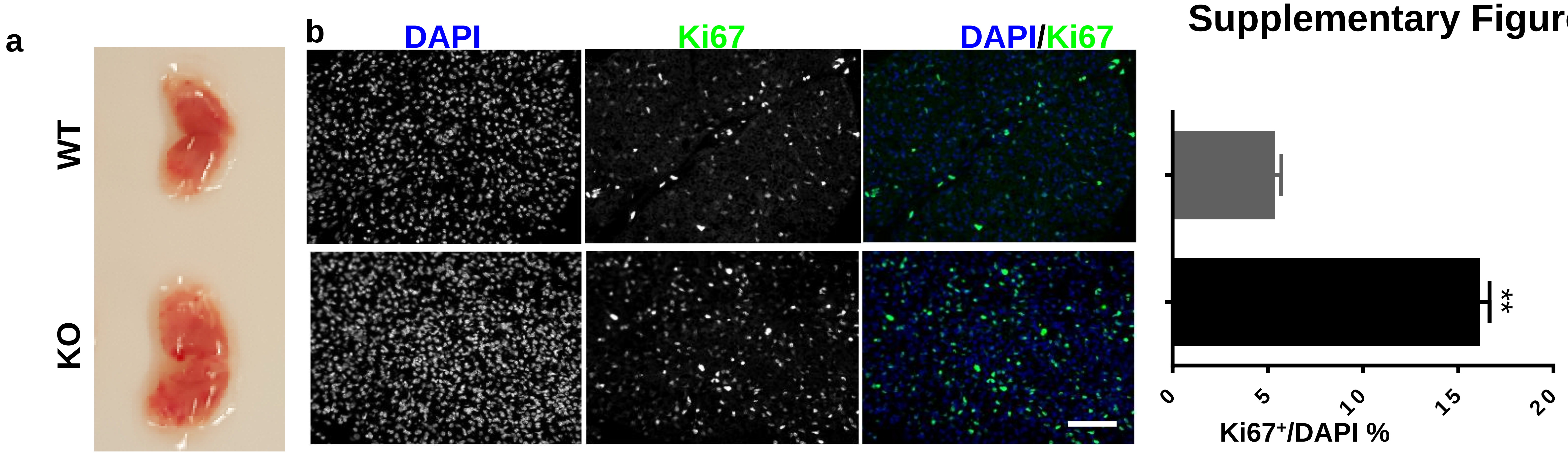

Supplementary Figure 2. Deletion of Lkb1 promotes BAT hyperplasia. (a-b) Representative BAT tissues (**a**) and Ki67 staining of BAT sections from P2 (postnatal day 2) WT and KO mice (**b**). The % $Ki67^+$ cells was shown in the right panel (n=4). Error bars: s.e.m. ** *P*< 0.01, two-tailed Student's t test. Scale bars: 100 µm .

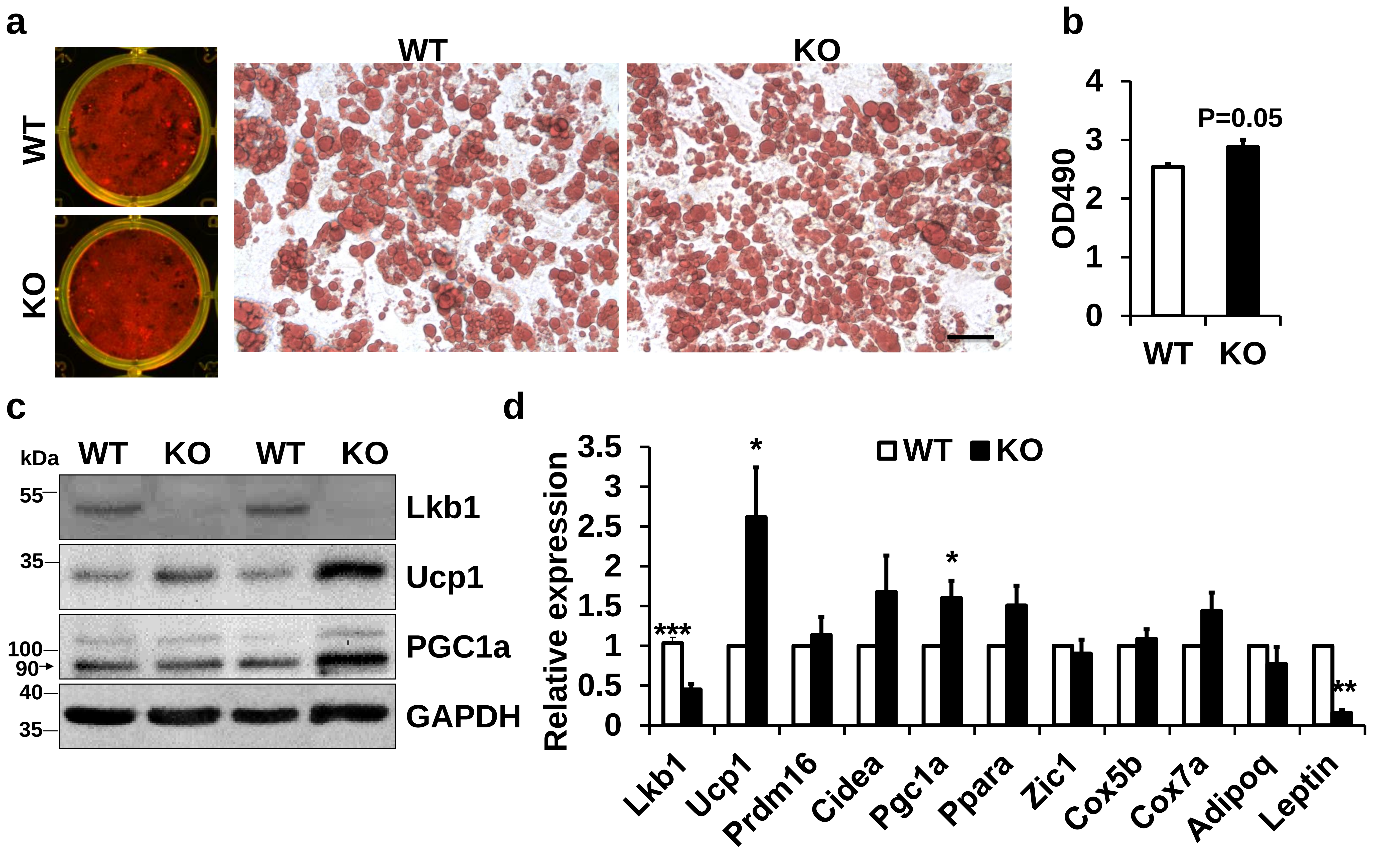

Supplementary Figure 3. Ablation of Lkb1 increases expression of brown adipose specific genes in cultured adipocytes. (**a**) Oil red O staining of WT (*Lkb1*$^{flox/flox}$) and KO (*Adipoq-Lkb1*) BAT SVF cells after differentiation. (**b**) Triglyceride (TG) levels in WT and Lkb1-KO cells after differentiation. n=5. (**c**) Protein levels of Lkb1, Ucp1 and PGC1a in WT and Lkb1-KO cells after differentiation. (**d**) mRNA levels of adipose tissue related genes in WT and Lkb1-KO cells after differentiation (n=9). Error bars: s.e.m. * P< 0.05, ** P< 0.01, *** P< 0.001, two-tailed Student's t test. Scale bar: 100 µm.

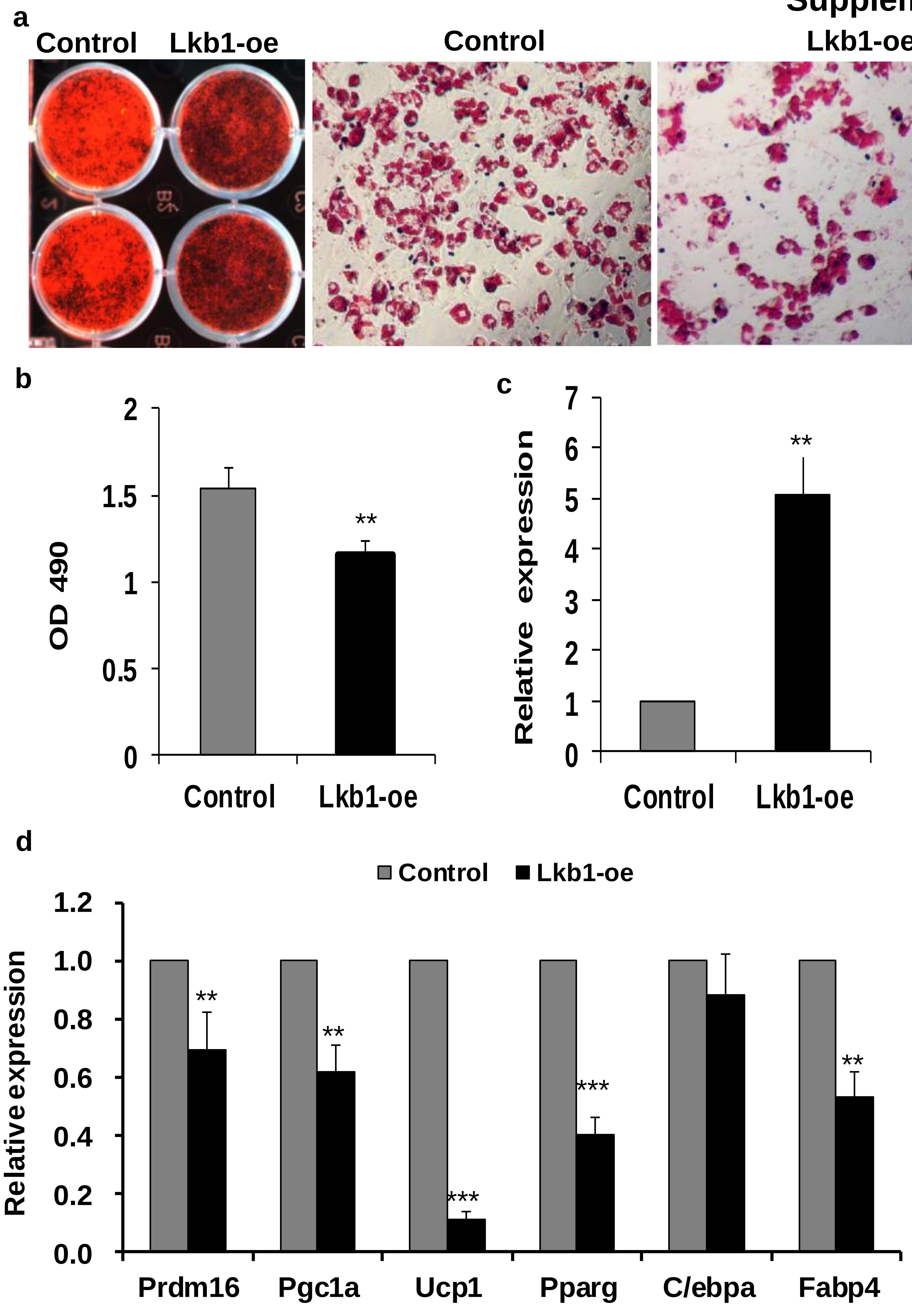

Supplementary Figure 4. Overexpression of Lkb1 inhibits lipid accumulation and expression of brown adipose specific genes in cultured adipocytes. (**a**) Oil red O staining of control and Lkb1 overexpression (Lkb1-oe) BAT SVF cells after differentiation. (**b**) Triglyceride (TG) levels were measured in control and Lkb1-oe cells based on OD490 (n=4). (**c, d**) Relative mRNA levels of Lkb1 (**c**) and BAT signature genes (**d**) in control and Lkb1-oe cells (n=4). Error bars: s.e.m. ** *P*< 0.01, *** *P*< 0.001, two-tailed Student's t test. Scale bar: 100 μm.

Supplementary Figure 5

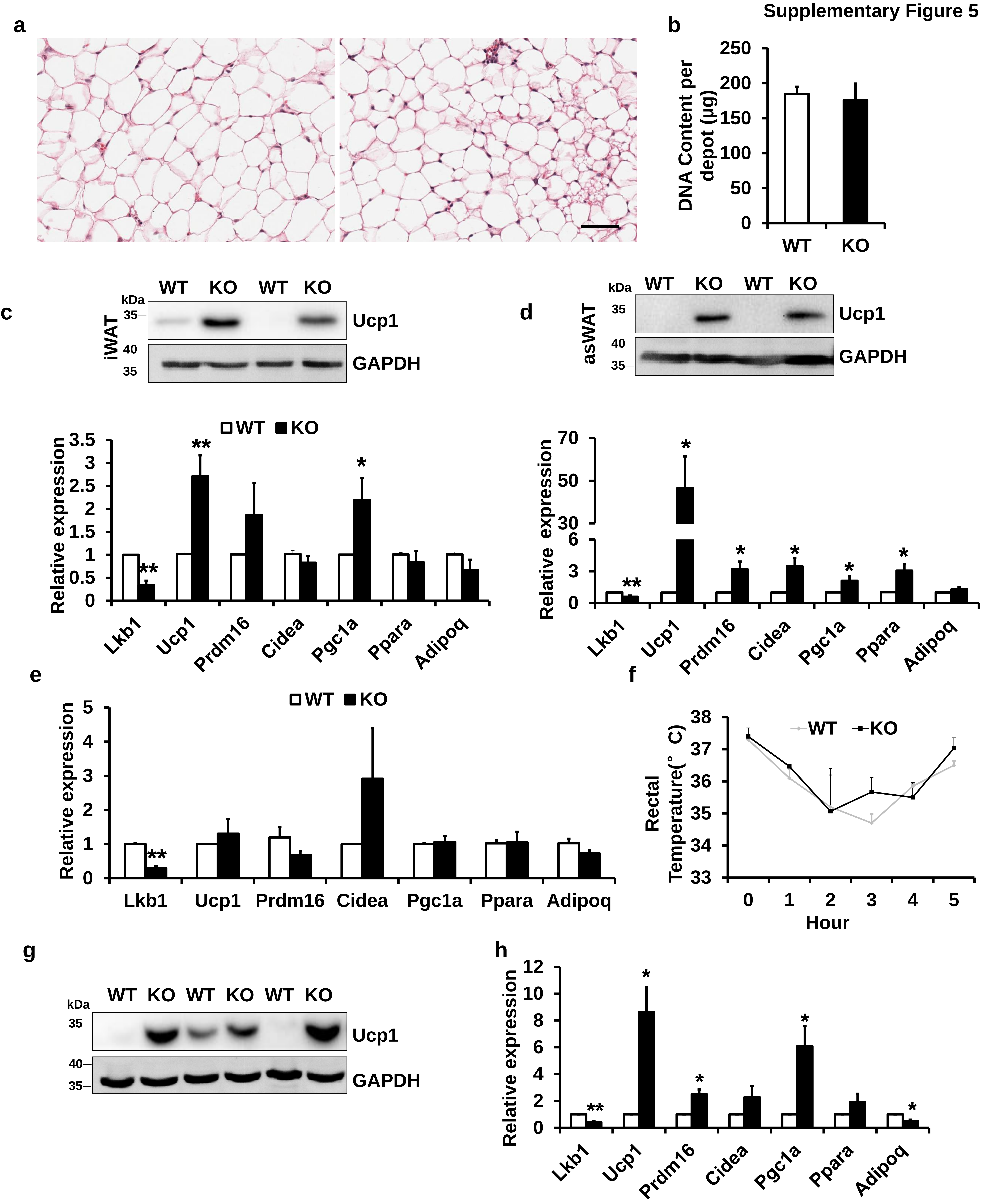

Supplementary Figure 5. **Ablation of Lkb1 promotes browning of subcutaneous WAT** (**a**) Hematoxylin and eosin (H&E) staining of iWAT sections from WT and KO at 10-week-old. (**b**) Genomic DNA content per fat depot from 6-wk-old male WT (n=5) and KO (n=4) mice. (**c, d**) Relative mRNA levels of brown fat selective genes in iWAT (**c**) and asWAT (**d**) from 4-6 week-old mice (n=8). Insets are Western blots showing Ucp1 protein levels in iWAT and asWAT. (**e**) Relative mRNA levels of brown fat related genes in eWAT WT and KO mice (n=4). (**f**) Rectal temperature of WT and KO male mice after cold stimulation (n=4). (**g**) Relative protein levels of Ucp1 in iWAT from WT and KO male mice (8-week-old) maintained at 4 ºC for 6 h. (**h**) Relative mRNA levels of browning marker genes in iWAT from WT and KO male mice (8-week-old) maintained at 4 ºC for 6 h (n=4). Error bars: s.e.m. * *P*< 0.05, ** *P*< 0.01, two-tailed Student's t test. Scale bars: 100 μm.

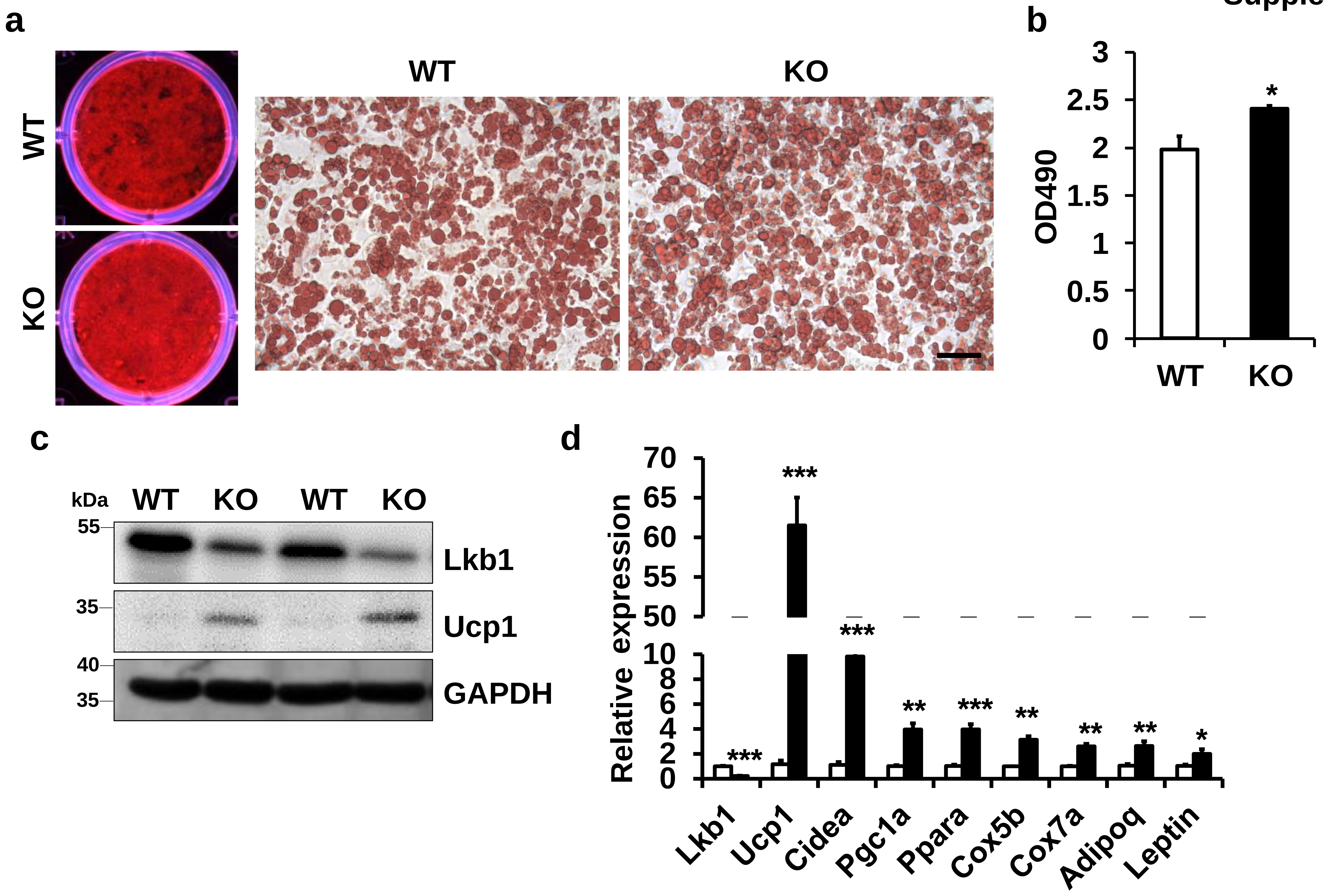

Supplementary Figure 6. Deletion of Lkb1 promotes browning of cultured white adipocytes. (**a**) Oil red O staining of WT and KO iWAT SVF cells after differentiation. (**b**) Triglyceride (TG) levels based on OD490 (WT n=4, KO n=5). (**c**) Relative protein levels of Lkb1 and Ucp1 in WT and Lkb1-KO cells after differentiation. (**d**) Relative mRNA levels of adipogenesis related genes in WT and Lkb1-KO cells after differentiation (n=9). The Error bars: s.e.m. * *P*< 0.05, ** *P*< 0.01, *** *P*< 0.001, two-tailed Student's t test. Scale bars: 100 μm.

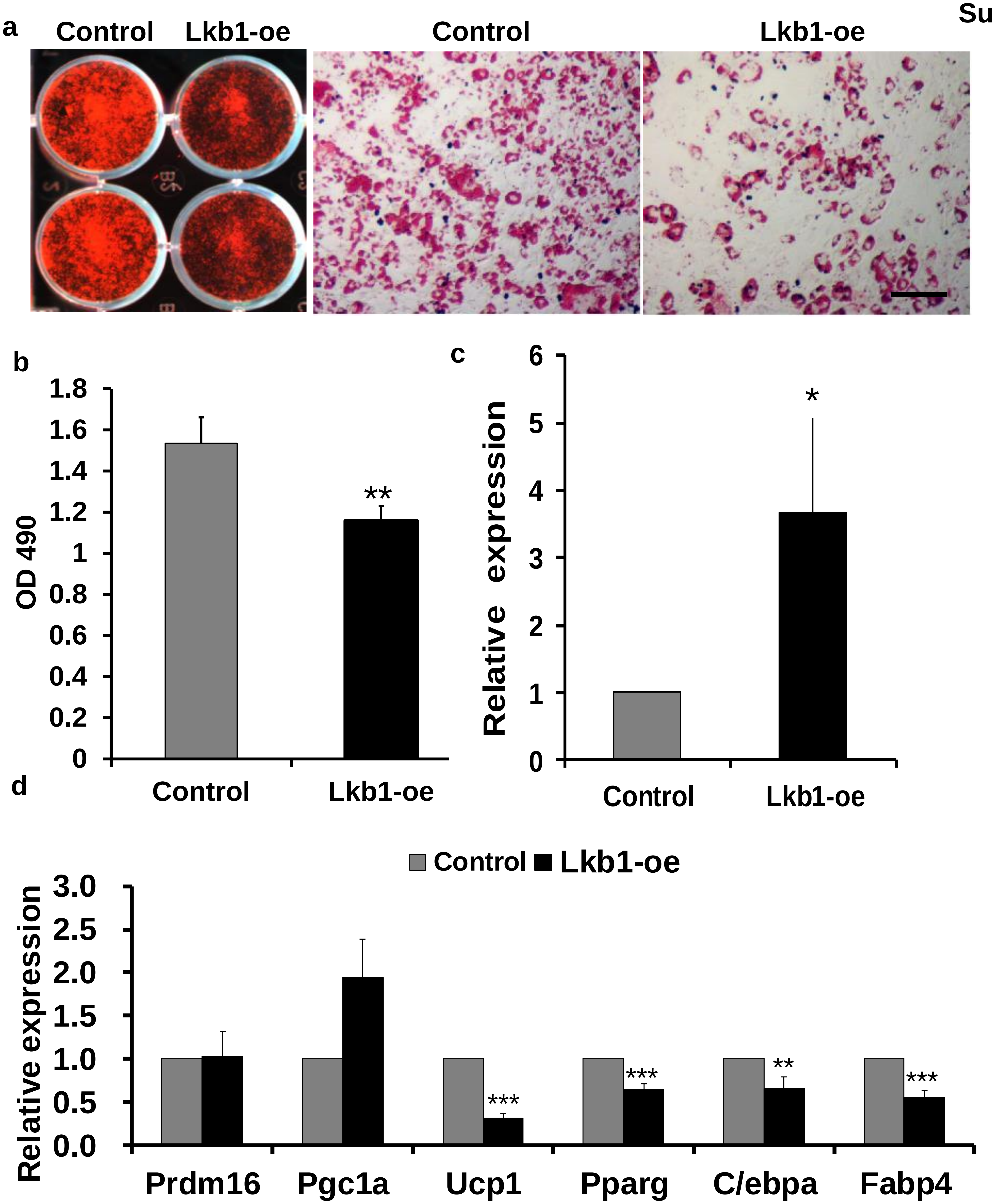

Supplementary Figure 7. Overexpression of Lkb1 inhibits white adipocyte differentiation and expression of adipogenic genes. (**a**) Oil red O staining of control and Lkb1 overexpression (Lkb1-oe) iWAT SVF cells after differentiation. (**b**) Triglyceride (TG) levels based on OD490 (n=4). (**c**, **d**) Relative mRNA levels of Lkb1(**c**), adipogenic and BAT signature genes (**d**), n=4. Error bars: s.e.m. ** P< 0.01, *** P< 0.001, two-tailed Student's t test. Scale bar: 100 µm.

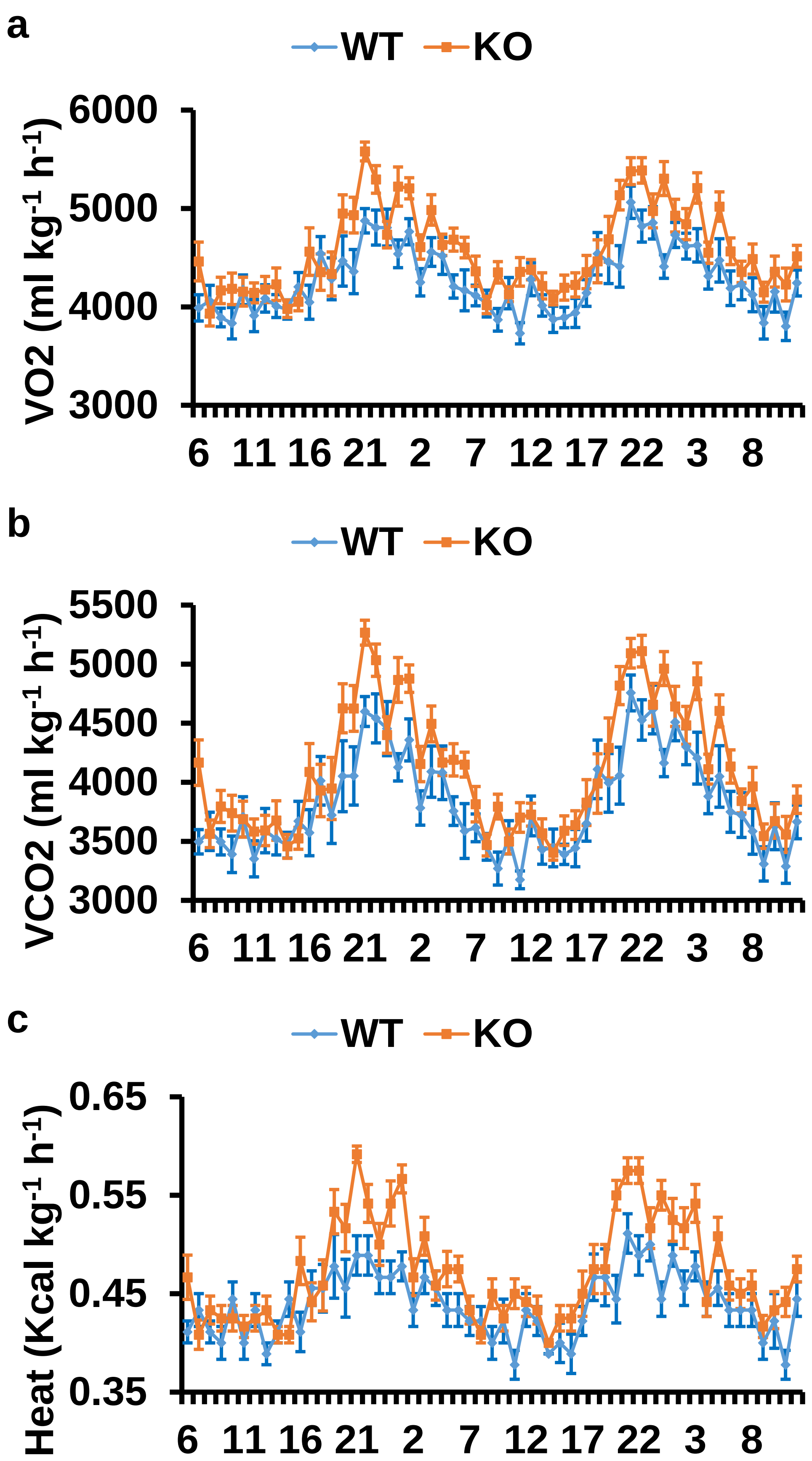

Supplementary Figure 8. Adipose specific deletion of Lkb1 in mice increases systemic energy expenditure. Oxygen consumption (VO2, **a**), carbon dioxide production (VCO2, **b**), and heat production (**c**) were measured using indirect calorimetry (WT n=3, KO n=4). Error bars: s.e.m.

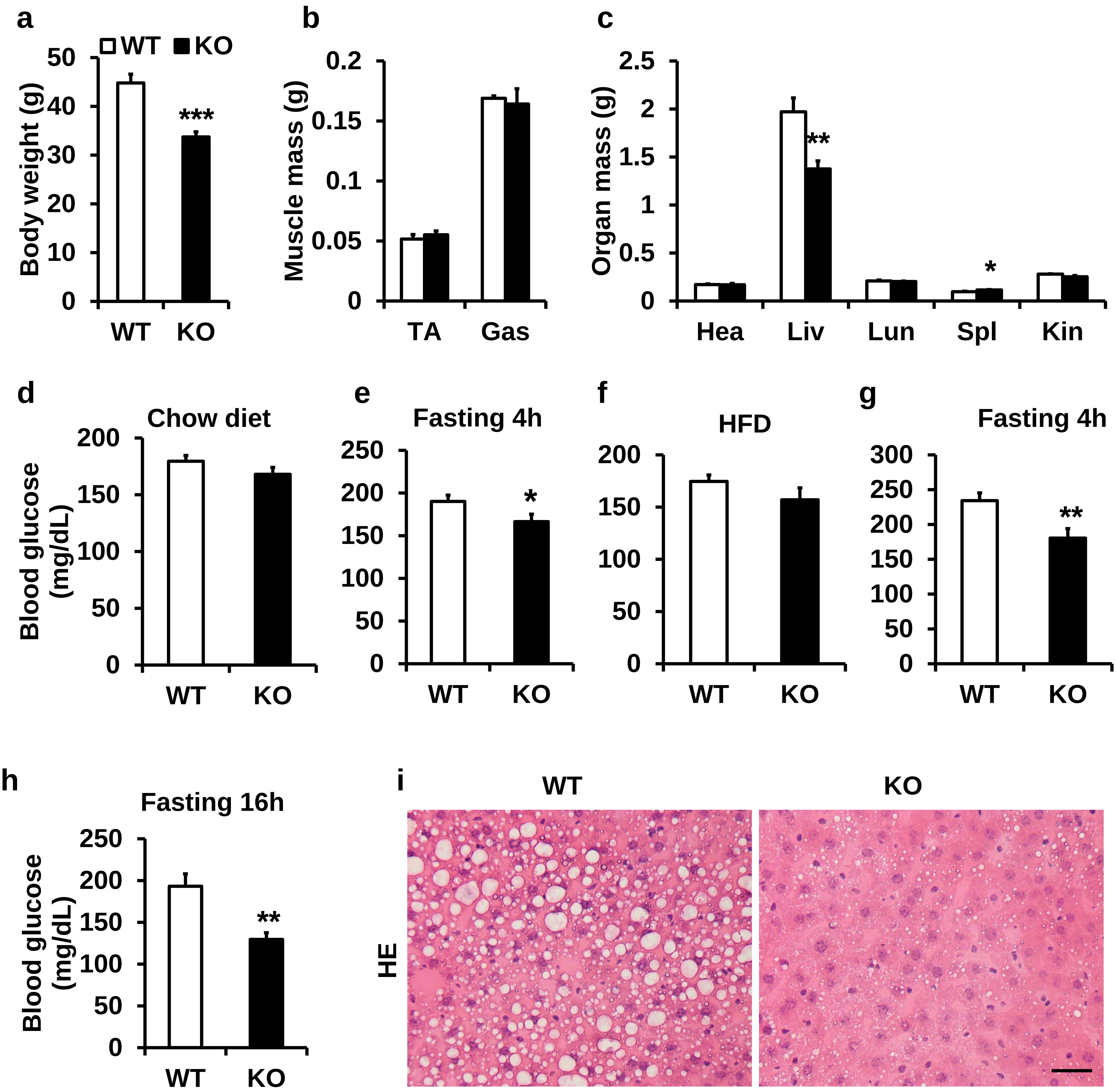

Supplementary Figure 9. Adipose specific deletion of Lkb1 improves glucose metabolism and protects high fat diet (HFD)-induced obesity. (**a**) Body weight of the WT (n=6) and KO (*Adipoq-Lkb1*, n=7) mice after 12 weeks on HFD. (**b, c**) Muscle (**b**) and organ (**c**) mass of the WT (n=6) and KO (n=7) mice after 12 weeks HFD. (**d, e**) Blood glucose levels of the 10-week old WT and KO mice fed on chow diet (**d**, WT n=6, KO n=4) or after fasting 4 h (**e**, n=8). (**f-h**) Fed (**f**, WT n=5, KO n=7) and fasted (**g**, 4 h, WT n=9, KO n=10; **h**, 16h, WT n=8, KO n=9) blood glucose levels of the WT and KO mice after 10 weeks HFD. (**i**) H&E staining of liver sections of WT and KO mice after 12 weeks HFD. Error bars: s.e.m. * $P < 0.05$, ** $P < 0.01$, *** $P < 0.001$, two-tailed Student's t test. Scale bars: 100 μm

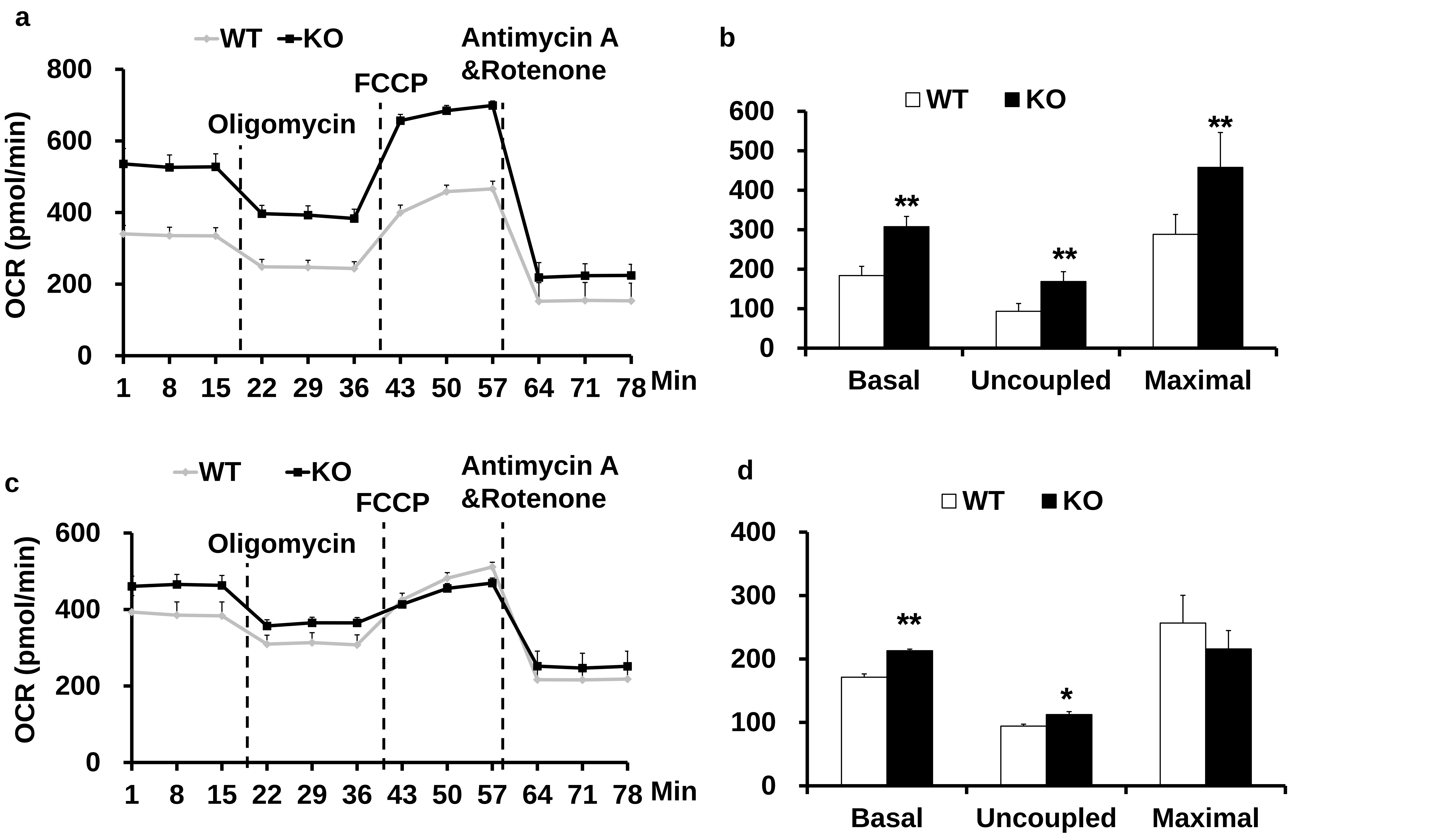

Supplementary Figure 10. Oxygen consumption rate (OCR) of adipocytes differentiated from WT and Lkb1 KO SVF cells. (**a**) OCR of BAT adipocytes measured at 7-min intervals with sequential addition of Oligomycin, FCCP and Antimyicin A/Rotenone to determine the uncoupled, maximal and non-mitochondrial mediated OCR, respectively (n=7). (**b**) Average basal, uncoupled and maximal OCR after subtracting non-mitochondrial mediated OCR in BAT adipocytes. (**c**) OCR of iWAT adipocytes measured as in **a** (n=3). (**d**) Average basal, uncoupled and maximal OCR after subtracting non-mitochondrial mediated OCR in WAT adipocytes. Error bars: s.e.m. * *P*< 0.05, ** *P*< 0.01, two-tailed Student's t test.

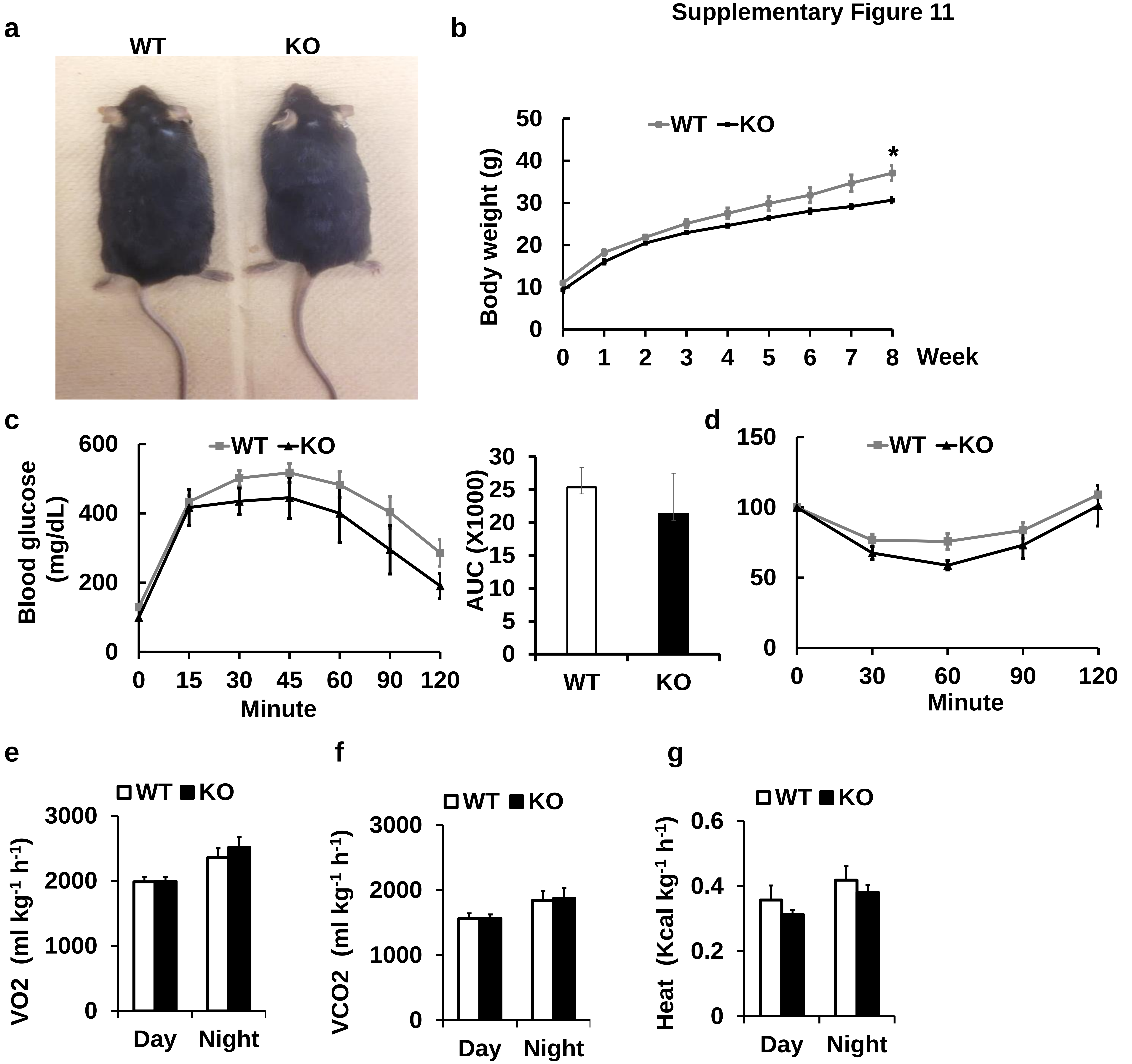

Supplementary Figure 11. **Deletion of Lkb1 affects glucose metabolism depending on Ucp1 function.** WT and KO mice were housed at thermoneutrality (30 ° C) to block Ucp1 function and fed with a high-fat diet for 10 weeks. (**a**) Representative images of WT and KO mice after treatment. (**b**) Growth curve of WT and KO mice (n=4). (**c, d**) Blood glucose concentrations during GTT (**c**, WT n=6, KO n=4) and ITT (**d**, WT n=6, KO n=4) after treatment. (**e-g**) Average day and night O2 consumption (VO2, **e**), CO2 production (VCO2, **f**) and heat production (**g**) (WT n=6, KO n = 4) after treatment. Error bars: s.e.m. * *P*< 0.05, ** *P*< 0.01, two-tailed Student's t test.

Supplementary Figure 12

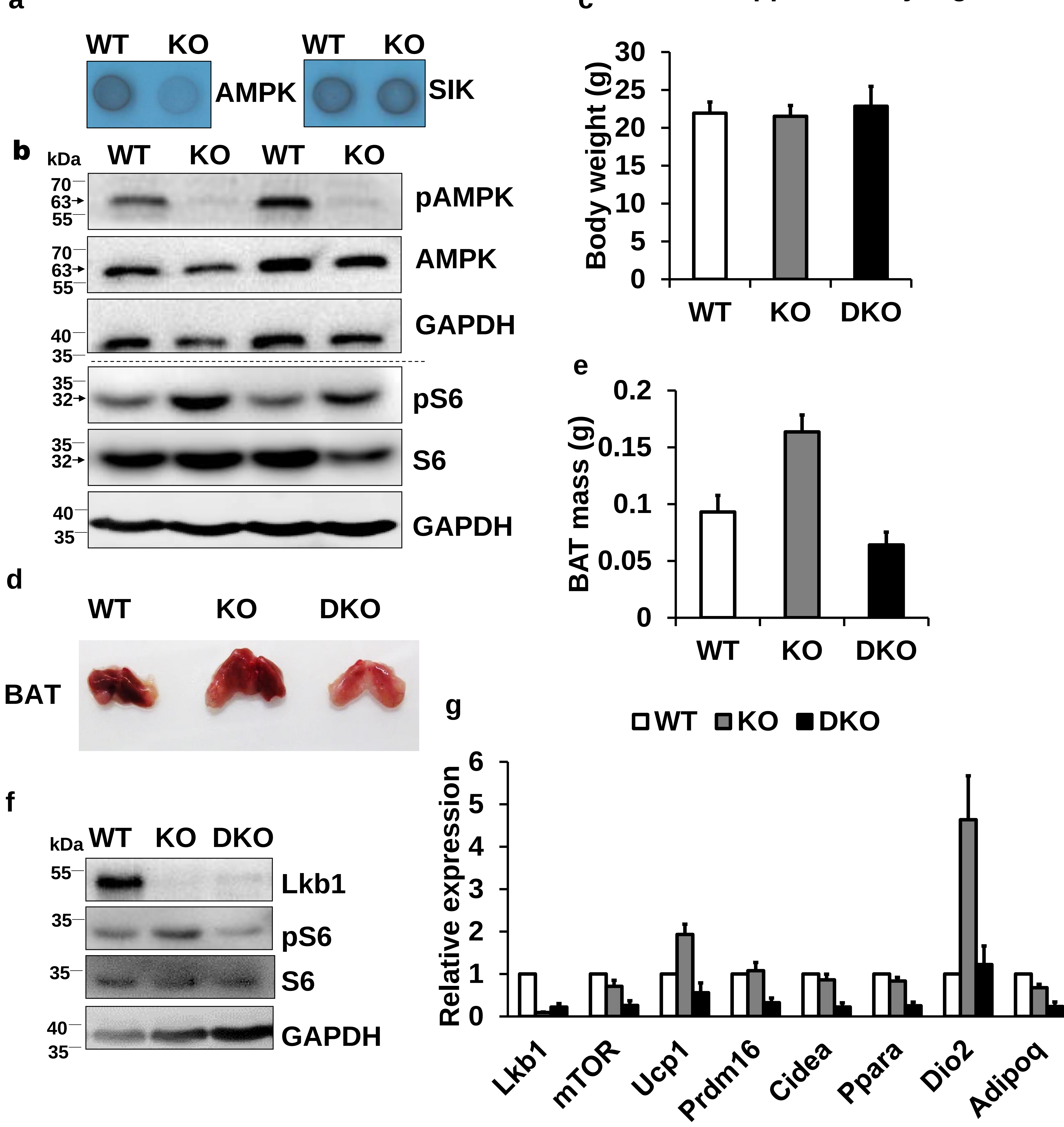

Supplementary Figure 12. Deletion of Lkb1 promotes BAT expansion through mTOR signaling pathway. (**a**) Relative kinase activity of AMPK and SIK immunoprecipitated from BAT of WT (*Lkb1*$^{flox/flox}$) and KO (*Adipoq-Lkb1*) mice. (**b**) Relative Protein levels of pAMPK, AMPK, pS6 and S6 in BAT from WT and KO mice (n=4). (**c**) Body weights of WT (n=5), KO (n=6) and DKO (*Adipoq-Lkb1-mTOR, n=3*) mice at 7-week old. (**d**) Representative images of BAT from WT, KO and DKO mice. (**e**) BAT mass of WT (n=5), KO (n=6) and DKO (*n=3*) mice at 7-week old. (**f**) Protein levels of Lkb1, S6 and pS6 in BAT of WT, KO and DKO mice. (**g**) Relative mRNA levels of Ucp1 and BAT related genes in BAT of WT (n=5), KO (n=6) and DKO (*n=3*) mice at 7-week old. Error bars: s.e.m.

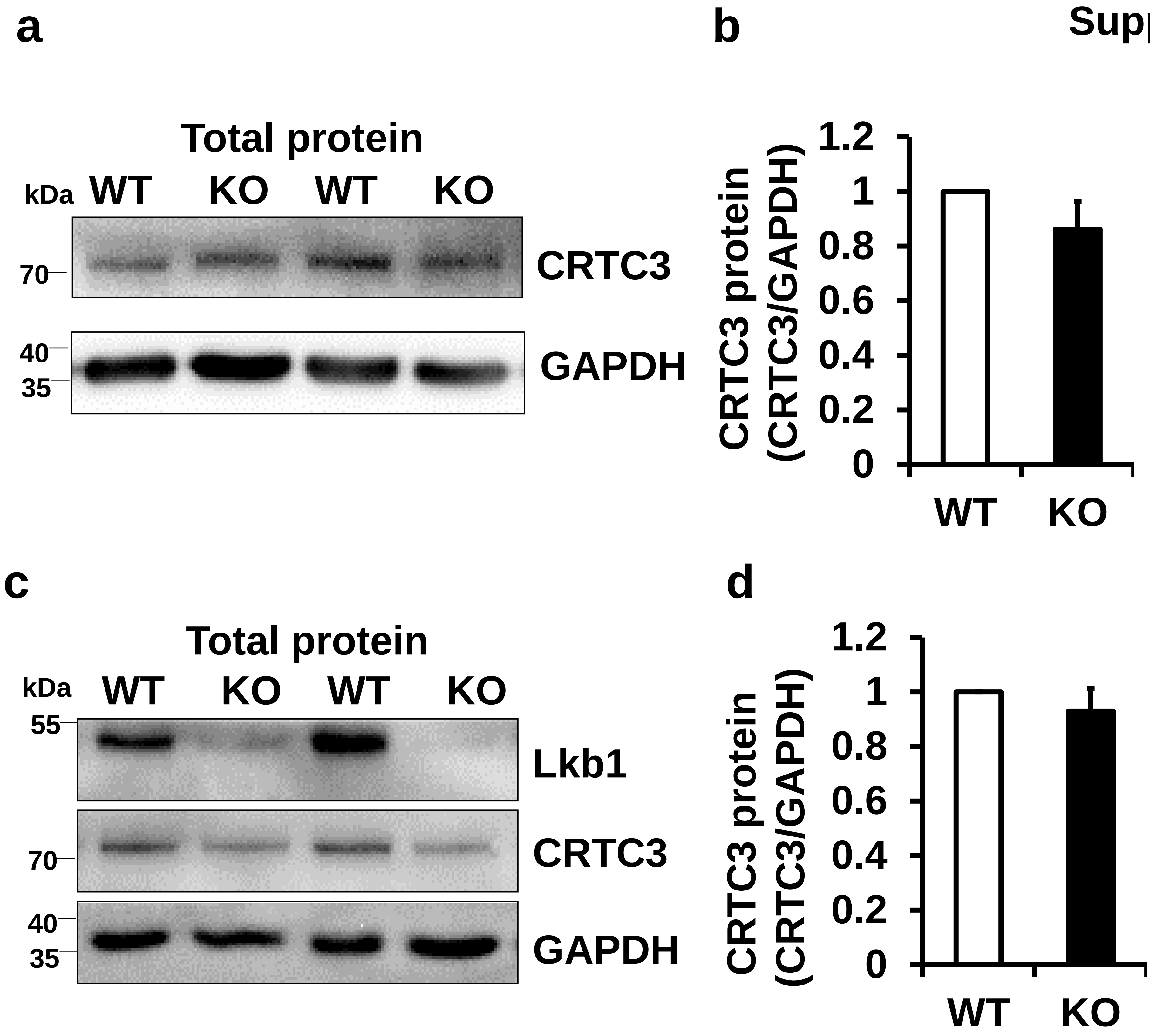

Supplementary Figure 13. Adipose specific ablation of Lkb1 does not affect overall expression of CRTC3. (**a, b**) Relative protein levels of CRTC3 in BAT of WT and KO (*Adipoq-Lkb1*) mice. GAPDH were used as control for western blot analysis and quantification (n=8). (**c, d**) Quantitative protein level of CRTC3 in differentiated BAT adipocytes measured by densitometry (n=8).

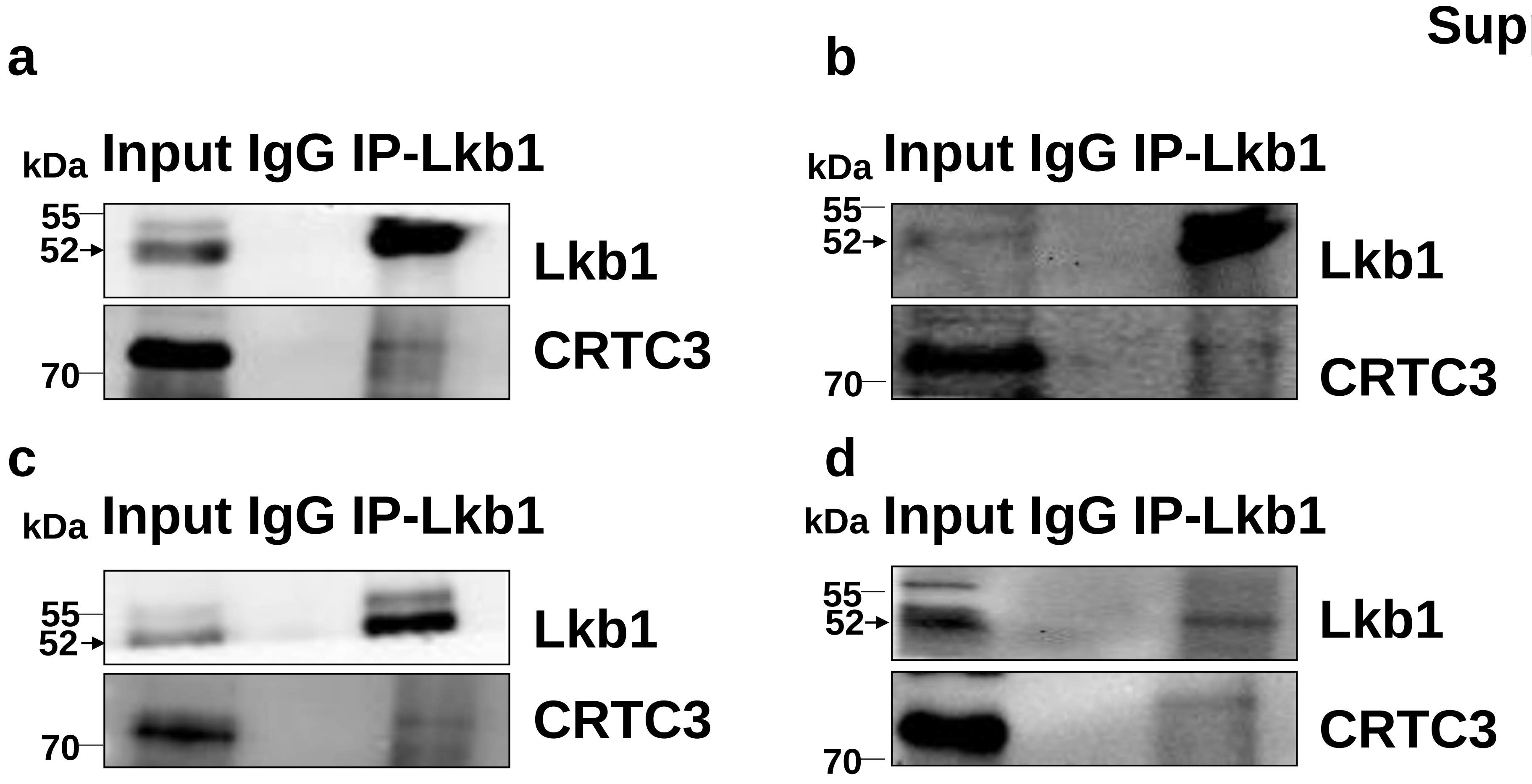

Supplementary Figure 14. Endogenously expressed Lkb1 and CRTC3 interact with each other. Cell lysates from differentiated brown adipocyte (**a**), 3T3-L1 cells (**b**), BAT (**c**) and iWAT (**d**) were immunoprecipitated (IP) with Lkb1 antibody, and blotted with Lkb1 and CRTC3 antibodies.

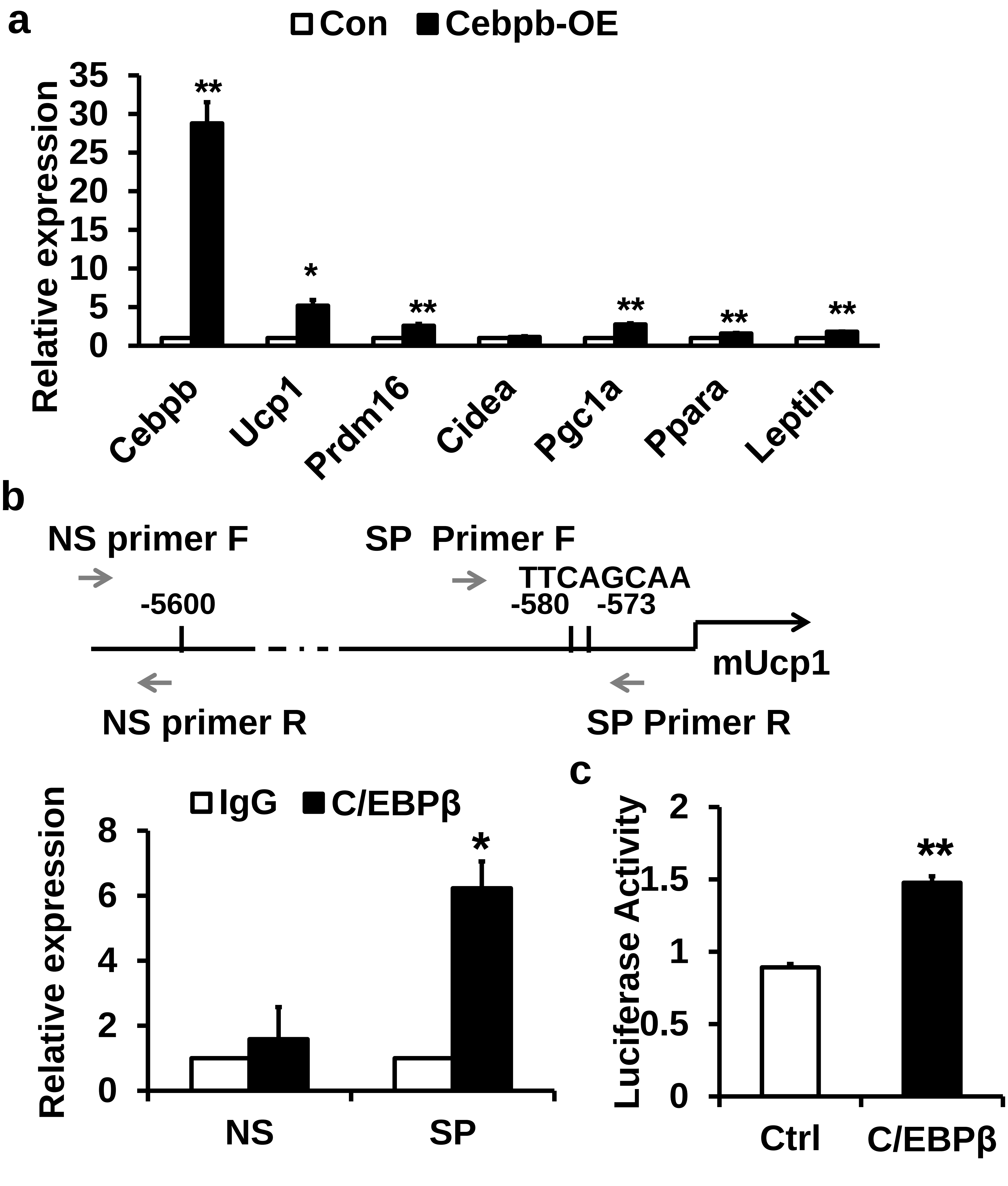

Supplementary Figure 15. C/EBPβ regulates Ucp1 expression. (**a**) Overexpression of C/EBPβ in BAT cells increases the expression of brown fat related genes. (**b**) Chromatin immunoprecipitation using a C/EBPβ specific antibody followed by qPCR amplified by primers flanking C/EBPβ specific (SP) DNA sequence or non-specific (NS) sequences in the promoter region of Ucp1 gene (n=3). (**c**) Luciferase assay of 293T cells after co-transfection of pGL3-Ucp1 plasmid and pcDNA-C/EBPβ (or control vectors) (n=8). Error bars: s.e.m. * $P < 0.05$, ** $P < 0.01$, two-tailed Student's t test.

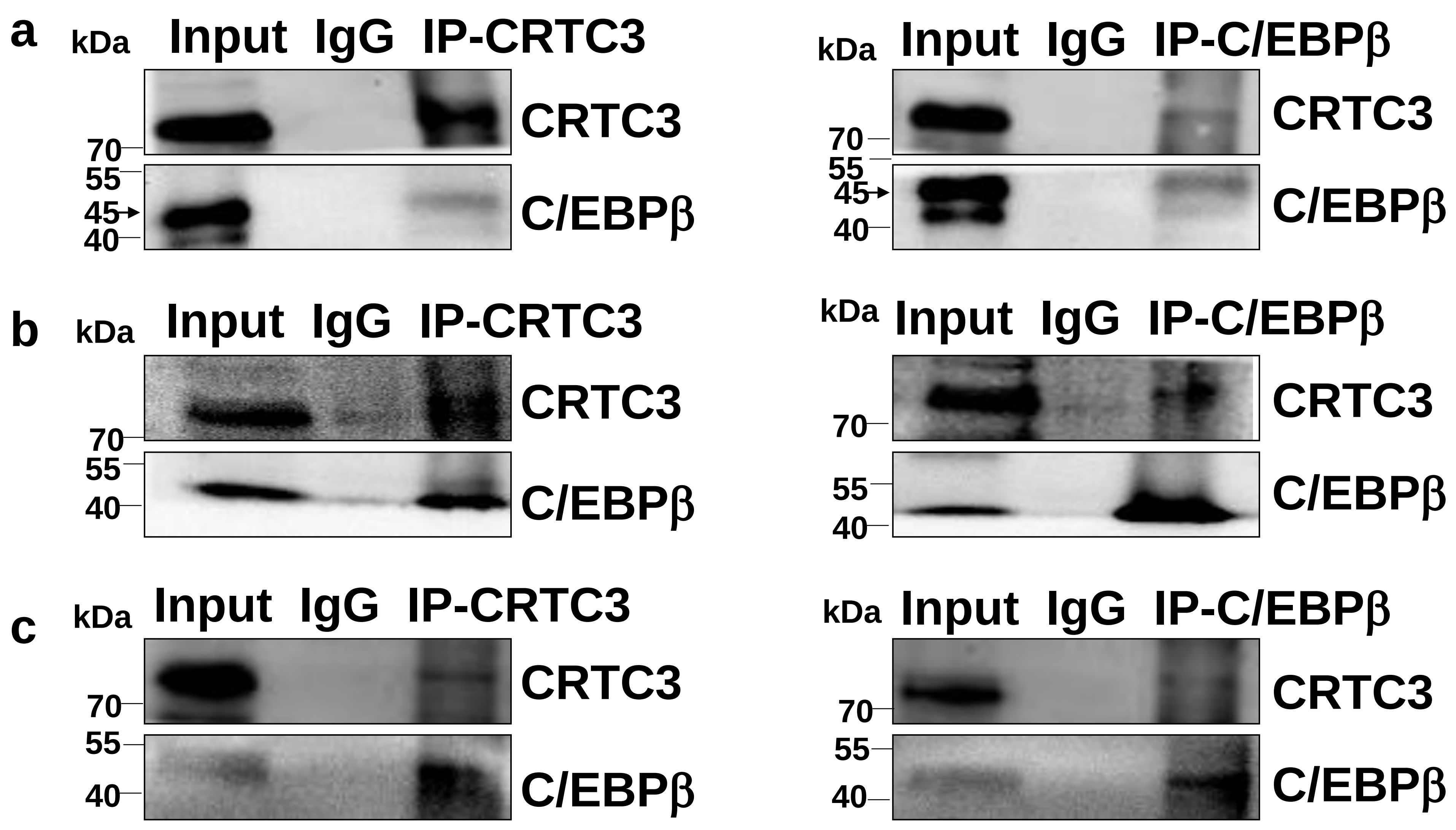

Supplementary Figure 16. Endogenously expressed CRTC3 and C/EBPβ interact with each other. Cell lysates from differentiated brown adipocyte (**a**), 3T3-L1 cells (**b**) and BAT(**c**) were immunoprecipitated (IP) with CRTC3 or C/EBPβ antibodies, and blotted with CRTC3 or C/EBPβ antibodies.

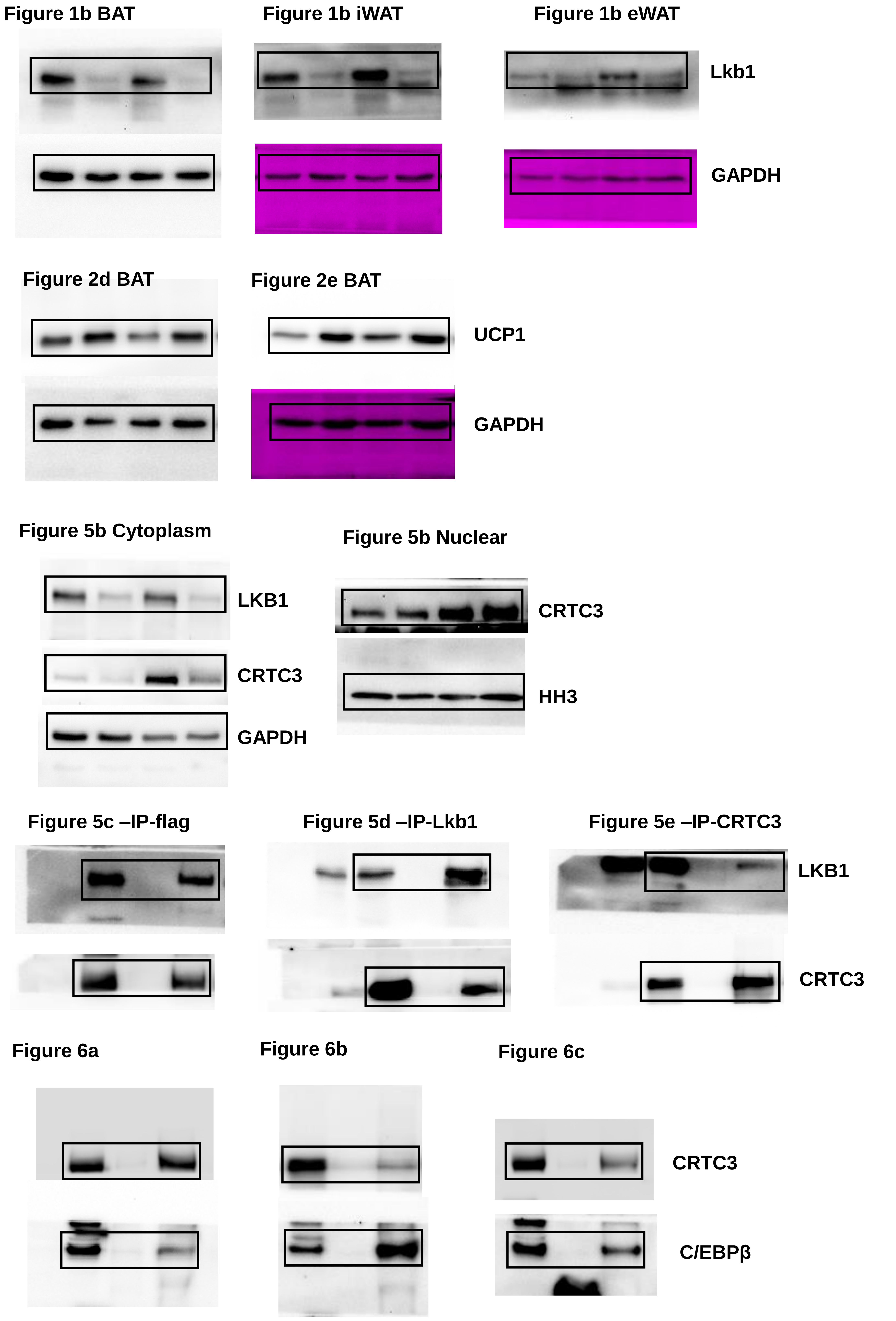

Supplementary Figure 17 Uncropped blots of main figures.

Supplementary Table 1 The primer sequence of real-time PCR

Primer name	Sequence (5' -> 3')	Temperature (°C)	Amplicon (bp)
Lkb1 F	TTGGGCCTTTTCTCCGAGG		
Lkb1 R	CAGGTCCCCCATCAGGTACT	61	138
Ucp1 F	AGGCTTCCAGTACCATTAGGT		
Ucp1 R	CTGAGTGAGGCAAAGCTGATTT	60	133
Prdm16 F	CCACCAGCGAGGACTTCAC		
Prdm16 R	GGAGGACTCTCGTAGCTCGAA	62	107
Cidea F	TGACATTCATGGGATTGCAGAC		
Cidea R	GGCCAGTTGTGATGACTAAGAC	60	171
Dio2 F	AATTATGCCTCGGAGAAGACCG		
Dio2 R	GGCAGTTGCCTAGTGAAAGGT	60	125
Pgc1a F	TATGGAGTGACATAGAGTGTGCT		
Pgc1a R	CCACTTCAATCCACCCAGAAAG	60	134
Ppara F	AGAGCCCCATCTGTCCTCTC		
Ppara R	ACTGGTAGTCTGCAAAACCAAA	60	153
Cox5b F	TTCAAGGTTACTTCGCGGAGT		
Cox5b R	CGGGACTAGATTAGGGTCTTCC	60	238
Cox7a F	GCTCTGGTCCGGTCTTTTAGC		
Cox7a R	GTACTGGGAGGTCATTGTCGG	60	100
Pparg F	TCGCTGATGCACTGCCTATG		
Pparg R	GAGAGGTCCACAGAGCTGATT	60	103
Adipoq F	TGTTCCTCTTAATCCTGCCCA		
Adipoq R	CCAACCTGCACAAGTTCCCTT	60	104

Leptin F	GAGACCCCTGTGTCGGTTC		
Leptin R	CTGCGTGTGTGAAATGTCATTG	60	139
Agt F	TCTCCTTTACCACAACAAGAGCA		
Agt R	CTTCTCATTCACAGGGGAGGT	60	121
Retn F	AAGAACCTTTCATTTCCCCTCCT		
Retn R	GTCCAGCAATTTAAGCCAATGTT	60	167
Trim14 F	GTGCGTGTGCAGAAGCTAATC		
Trim14 R	CTGCGTAAACCTTGAGCCTTT	60	118
Zic1 F	CAGTATCCCGCGATTGGTGT		
Zic1 R	GCGAACTGGGGTTGAGCTT	62	139
Fabp4 F	AAGGTGAAGAGCATCATAACCCT		
Fabp4 R	TCACGCCTTTCATAACACATTCC	61	133
mTOR F	ACCGGCACACATTTGAAGAAG		
mTOR R	CTCGTTGAGGATCAGCAAGG	60	110

Supplementary Movie 1. Movement of WT (left) and *Adipoq-Lkb1* (right) mice in the new cages.
